# High diversity but limited expression of biosynthetic gene clusters in a peatland microbial community

**DOI:** 10.1038/s41467-026-78121-8

**Published:** 2026-10-06

**Authors:** Zander Rainier Human, Rene Hoover, Clara Chan, Kirsten Küsel, Carl-Eric Wegner

**Affiliations:** 1 Institute of Biodiversity, Ecology & Evolution, Aquatic Geomicrobiology, Friedrich Schiller University Jena, Jena, Germany; 2 Department of Earth Sciences, University of Delaware, Newark, DE USA; 3 Microbiology Graduate program, University of Delaware, Newark, DE USA; 4 Cluster of Excellence Balance of the Microverse, Friedrich Schiller University Jena, Jena, Germany; 5 German Center for Integrative Biodiversity Research (iDiv) Halle-Jena-Leipzig, German Center for Integrative Biodiversity Research (iDiv) Halle-Jena-Leipzig, Leipzig, Germany; 6 Department of Inorganic Chemistry, Bioinorganic Chemistry, Heinrich Heine University Düsseldorf, Düsseldorf, Germany

**Keywords:** Microbial ecology, Soil microbiology, Metagenomics

## Abstract

The in situ relevance of biosynthetic gene clusters (BGCs) remains poorly understood. We apply meta-omics to characterize BGC diversity and activity along a peatland redox gradient. From seven metagenomes, we recover 9,694 BGCs, spanning diverse taxa, most lacking close relatives in reference databases, indicating extensive novelty. Only 9-27% of this potential is expressed in situ, with Acidobacteriota, despite moderate repertoires, accounting for over half of all BGC transcription. Talented producers with up to 24 clusters are largely silent, and expression is inversely related to BGCs per genome. Acidobacteriota, specialized in complex carbon processing potential express a larger proportion of their BGC repertoires than BGC-rich Pseudomonadota, which had shorter doubling times and lower potential for complex carbon processing. At the genome level, the degree of BGC expression is most strongly coupled to carbohydrate-active enzyme expression, particularly glycoside hydrolases, linking secondary metabolism to active carbon processing in peatland microbes. Thus, although BGCs are widespread, BGC expression in situ is integrated into the carbon-cycling activity of individual taxa rather than a shared physiological state.

## Abstract (web summary)

Secondary metabolites, including metal chelators and antibiotics, are synthesized by diverse biosynthetic pathways. Here authors find that peatlands harbour extensive biosynthetic novelty, but their activation is only triggered by select conditions.

## Introduction

Secondary metabolites are synthesized by diverse biosynthetic pathways encoded by biosynthetic gene clusters (BGCs), which are groups of colocalized and coregulated genes. These metabolites are functionally diverse and include metal chelators, antifungals, and antibiotics^1–5^. The biosynthesis of secondary metabolites is energetically costly, and most known producers are aerobes^6^. This has contributed to the view that secondary metabolism is favoured in energy-rich, oxic environments. At the same time, environmental triggers and physiological transitions often provide the cues that activate secondary metabolite production in culture, including nutrient limitation^7,8^, especially nitrogen and phosphorus depletion^9^, and oxidative stress^10^. In *Streptomyces*, where most BGCs remain silent under standard growth conditions, biosynthesis is commonly induced by nutrient depletion, the quality of carbon sources, entry into the stationary phase, or cell-to-cell interactions^11,12^. Similar regulatory patterns are observed in other soil bacteria, such as *Burkholderia* and *Pseudomonas*, which produce secondary metabolites typically during stationary phase or under nutrient limitation^13^. In contrast, some myxobacteria, such as *Sorangium* spp., express nonribosomal peptide synthetase (NRPS) and polyketide synthase (PKS) clusters during exponential growth^14^. Across these lineages, the conditions that activate biosynthesis differ substantially, indicating that secondary metabolism is governed by lineage-specific physiological cues rather than a single shared trigger. Yet the evidence underlying this view comes almost exclusively from cultivable organisms. The study of BGCs has historically been limited to organisms that can be readily cultivated, resulting in a focus on a restricted set of lineages, primarily Actinobacteria (Actinomycetota) and Proteobacteria (Pseudomonadota). Cultivation, however, represents a strong bottleneck: it excludes most environmental microbes, masks ecological drivers of secondary metabolism, and often fails to activate many BGCs under artificial growth conditions. Consequently, the diversity and ecological relevance of microbial secondary metabolism remain poorly understood.

Advances in metagenomics, particularly the reconstruction of metagenome-assembled genomes (MAGs), have expanded the microbial tree of life^15–17^ and uncovered vast BGC diversity across environments ranging from oceans to soils^18–21^. These surveys demonstrate that BGCs are nearly ubiquitous across microbial lineages, including those uncultured and taxonomically underrepresented. They also suggest that the synthesis and utilization of secondary metabolites strongly depend on the environment.

Peatlands represent a particularly promising but still underexplored ecosystem in this context. They are globally significant carbon reservoirs^22^ and harbor microbial communities that are phylogenetically diverse, taxonomically novel, and underrepresented in culture collections^23^. Many of these lineages, including Acidobacteriota, Verrucomicrobiota, Planctomycetota, and other poorly studied groups, have been shown to harbor rich biosynthetic potential, yet the ecological roles of these metabolites remain largely unknown^18,24–26^. Peatland hydrology creates steep vertical redox gradients, with oxic surface layers transitioning into permanently anoxic, water-saturated horizons, accompanied by shifts in nutrient availability and electron acceptor competition^27–29^. These gradients create environmental conditions that parallel the nutrient and redox cues known to induce secondary metabolism in cultured bacteria^9^.

We hypothesize that peatlands are not only reservoirs of biosynthetic novelty but also natural laboratories for testing how environmental heterogeneity filters from the broad repertoire of BGCs present in microbial genomes. Here, we test if BGCs, although widespread across peatland microbial lineages, including many beyond the classical producers, are selectively structured by redox conditions in terms of distribution and expression. By combining metagenomic and transcriptomic analyses, we assess both the diversity and in situ expression of BGCs along natural redox gradients and show how the environment selects from the universal biosynthetic potential of microbes.

## Results

### Metagenomes from an acidic, Fe-rich peatland

To capture microbial diversity and biosynthetic activity across the natural redox gradient of the Schlöppnerbrunnen fen, we performed genome-resolved metagenomic and metatranscriptomic analyses on peat collected between 5 and 30 cm depth. This interval spans the transition from the oxic surface layer to the anoxic, water-saturated layer, where moisture and microbial activity remain high. Samples were collected in 5 cm increments, with a second core taken 1 m away for replication.

**Fig. 1:**
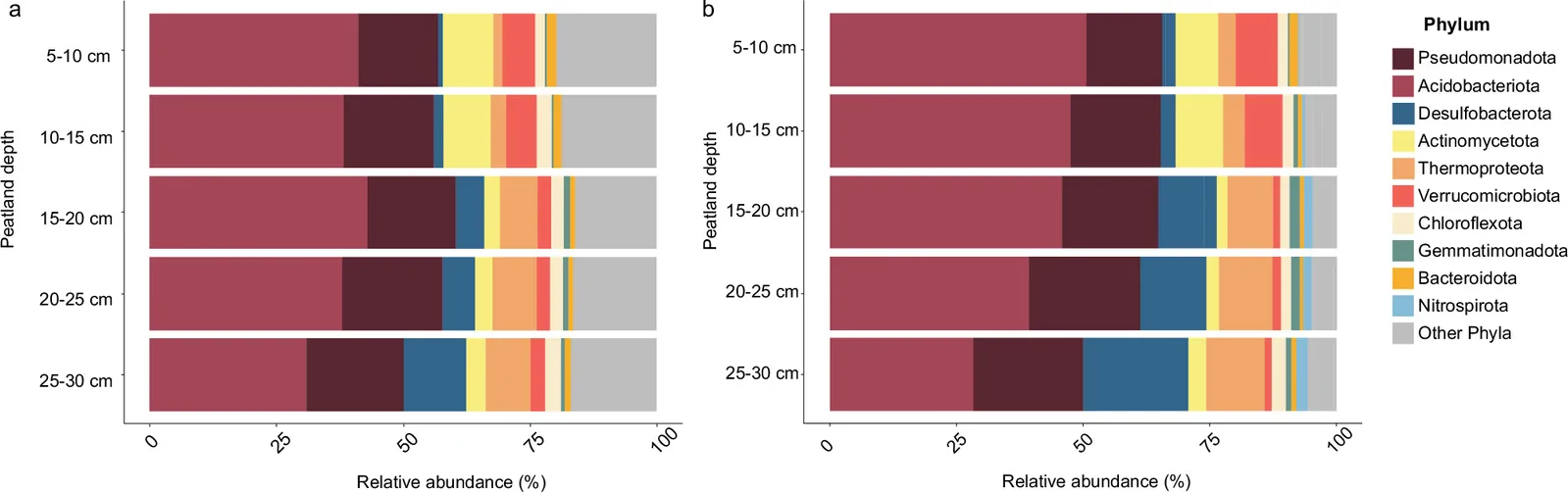
Microbial community composition and diversity across peatland depths. **a** Taxonomic profiles derived from raw reads assigned to single-copy marker genes using *SingleM* and **b** relative abundances of reconstructed MAGs, both shown as mean community composition across five depth intervals (5–30 cm). Source data are provided as a Source Data file.

Coverage estimations based on k-mer profiles confirmed that our deep sequencing adequately captured the genomic diversity of the peatland microbiome (Supplementary Table 1, Supplementary Figure 1). Across all depths, the peatland microbiome was dominated by members of the Acidobacteriota (30–43%), Pseudomonadota (16–19%), Actinomycetota (3–10%), Verrucomicrobiota (2.7–6.5%), and Patescibacteriota (5–11%) (Fig. 1a, Supplementary Table 2). The relative abundance of these phyla shifted markedly with depth. Acidobacteriota, Actinomycetota, and Verrucomicrobiota decreased in deeper layers, whereas Desulfobacterota, Archaea, and Pseudomonadota increased in abundance toward more anoxic conditions. This stratification reflects a transition from predominantly aerobic to anaerobic microbial assemblages along the redox gradient. Taxonomic composition was inferred from the abundance of single-copy housekeeping genes identified using *SingleM*^30^.

Metagenome assemblies were clustered into 573 metagenome-assembled genomes (MAGs) with at least 70% completeness and contamination less than 10% (Supplementary Data 1; Supplementary Fig. 2), exceeding the MIMAG standard for medium quality MAGs^31^ with a median completeness of 94.4 % and contamination of 2.2%. The majority of MAGs were assigned to Acidobacteriota (131 MAGs), Pseudomonadota (119), Actinomycetota (44), Verrucomicrobiota (37), and Desulfobacterota (43). From the 52 Archaea MAGs, most belonged to the Thermoproteota (31) and Halobacteriota (11) (Supplementary Table 3).

Relative MAG abundances, determined by mapping quality-controlled reads to MAGs with *CoverM*^32^, were consistent with taxonomic profiles obtained from raw reads using *SingleM*^30^, which assigns taxonomy based on single-copy marker genes (Fig. 1b, Supplementary Table 3). To assess how metabolic strategies varied along the redox gradient, MAG abundances were combined with O_2_-utilizing phenotype assignments predicted from genome-wide amino-acid k-mer composition^33^, and the number of oxygen-utilizing enzymes^34^. Both strategies indicated a clear increase in anaerobic taxa with depth, from 6 to 34% in the oxic surface layer to 29% to 50% in the deepest peat layer (25–30 cm depth) (Supplementary Fig. 3).

### Identification and abundance of BGCs

The biosynthetic potential of the peatland was assessed by identifying biosynthetic gene clusters (BGCs) from contigs longer than 5 kbp using *antiSMASH*^35^. A total of 9694 BGCs (Supplementary Table 4, Supplementary Data 2) were identified, 1594 (16.4%) were considered complete. The average length was 31,073 bp, the longest BGC was a 100,738 bp NRPS-Type I PKS-transAT-PKS-like cluster, and the shortest a 10,219 bp RiPP-like BGC. Because short-read assemblies can fragment large BGCs, especially modular NRPS and PKS clusters, we assessed the potential degree of inflation using the position of biosynthetic core genes relative to contig ends. Rather than discarding incomplete BGCs, we applied a filter focused on fragmentation-prone BGC classes. We flagged incomplete NRPS, PKS and NRPS-PKS hybrid BGCs lacking any biosynthetic core gene more than 5 kb from a contig end as weak fragments (Supplementary Data 3). Using this criterion, 1544 of 9694 BGCs (15.9%) were considered potential overestimates, which included 41.5% of NRPS BGCs, 39.2% of PKS BGCs and 20.5% of NRPS-PKS hybrid BGCs. Many contig-edge modular BGCs still retained anchored biosynthetic core genes and were therefore retained, indicating that incompleteness does not necessarily imply unreliability.

To evaluate the impact of assembly fragmentation on family-level diversity estimates, we combined the anchored-core filter with BiG-SCAPE^21^ GCF assignments. Excluding weak, highly fragmented BGCs reduced the total from 4669 to 3452 GCFs. Fragmentation of BGCs thus had a larger effect on GCF richness, with the removal of fragmented BGCs reducing total BGC counts by 16%, but GCF numbers by 26%. This likely reflects the contribution of small NRPS and PKS fragments to rare fragmentary GCFs. However, even after this conservative filtering, the peatland microbiome still comprised 3452 GCFs from only seven metagenomes. While such comparisons are not strictly equivalent across studies, owing to differences in sampling design and strictness of analyses thresholds, the observed diversity remains comparable in scale to that reported from much larger metagenomic surveys, often from tens to thousands of samples from the global ocean, Antarctic soil, rumen and agricultural soils dataset^19,36–38^.

**Fig. 2:**
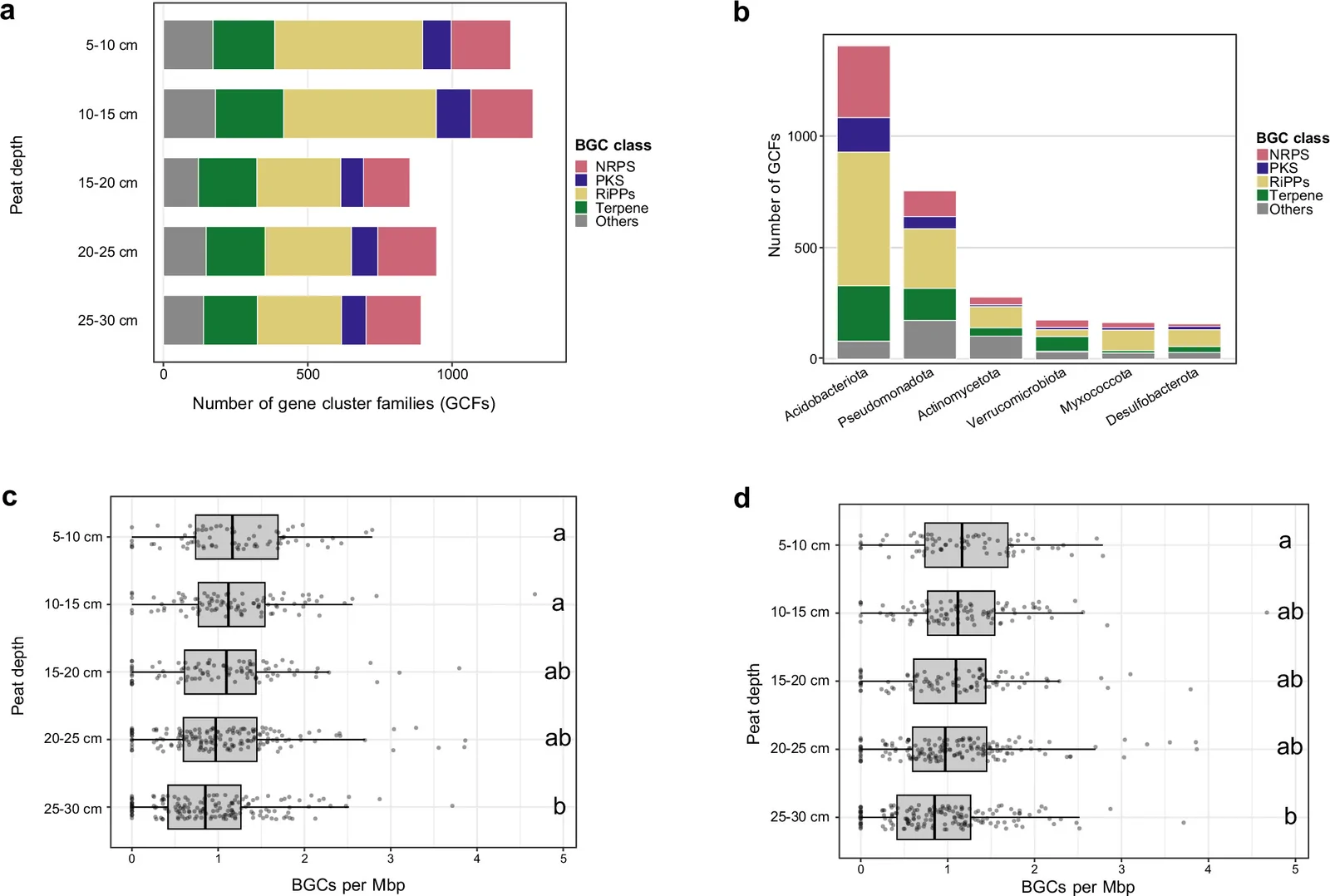
Diversity of biosynthetic gene clusters. **a** Distribution of BGC classes and their grouping into gene cluster families (GCFs). GCF groupings have been obtained through clustering 8150 fragment-filtered BGCs into families using BiG-SCAPE. **b** Number of GCFs assigned to taxa and peatland depth. **c** BGC density (BGCs per Mbp, calculated on estimated genome size), per MAG, among MAGs recovered from metagenomes representing five peat depth intervals. The statistical unit was the individual MAG, with *n* = 69, 89, 82, 166 and 164 MAGs for 5–10, 10–15, 15–20, 20–25 and 25–30 cm, respectively. **d** BGC density among Acidobacteriota MAGs, the most abundant phylum in this dataset, with *n* = 24, 27, 16, 32 and 32 MAGs at the same respective depths. Box plots show the median, 25th and 75th percentiles (box limits), and whiskers extending to the most extreme observations within 1.5× the interquartile range; observations beyond the whiskers are shown individually. Differences among the MAG-level distributions were assessed using Kruskal–Wallis tests. For all MAGs, BGC density differed among depth-associated MAG distributions (H(4) = 16.2, *P* = 0.00275, η²H = 0.0216, 95% CI [0.0052, 0.060]); for Acidobacteriota MAGs, H(4) = 12.8, *P* = 0.0125, η²H = 0.0695, 95% CI [0.0072, 0.220]. Pairwise comparisons were performed using two-sided Dunn tests with Benjamini–Hochberg adjustment for multiple comparisons. Significant comparisons were 5–10 versus 25–30 cm (*z* = −3.41, adjusted *P* = 0.00661) and 10–15 versus 25–30 cm (*z* = −3.03, adjusted *P* = 0.0123) in (**c**), and 5–10 versus 25–30 cm in (**d**) (*z* = −3.54, adjusted *P* = 0.00396). These analyses compare the distributions of recovered MAGs and do not constitute replicated tests of peat depth as an environmental treatment. Source data are provided as a Source Data file.

BGCs in the peatland microbiome encompassed a wide range of product classes, dominated by ribosomally synthesized and post-translationally modified peptides (RiPPs; *n* = 1361), terpenes (*n* = 620), nonribosomal peptide synthetases (NRPS; *n* = 510), and polyketide synthases (PKS; *n* = 367), and 107 NRPS-PKS hybrids (Fig. 2a; Supplementary Table 5). Within the RiPP category, the most abundant subtypes were RiPP-like clusters (531), RRE (RiPP recognition element)-containing clusters (333), ranthipeptides (194), lassopeptides (87), redox cofactors (52), linear azol(in)e-containing peptides (LAP; 39), thioamitides (30), and class IV lanthipeptides (73). Most RiPP subclasses are typically associated with terrestrial bacteria, although LAPs, lassopeptides, and lanthipeptides also occur in marine and freshwater taxa^39^.

Gene-cluster families (GCFs) were taxonomically widespread, dominated by Acidobacteriota (1404) and Pseudomonadota (754), followed by Actinomycetota (278), Verrucomicrobiota (175) and Myxococcota (164). Additional GCFs were contributed by Desulfobacterota (158), Planctomycetota (66), Nitrospirota (58), Bacteroidota (31) and Chloroflexota (28) (Fig. 2b). Archaeal GCFs were also present (*n* = 40), with roughly half (21) being of Thermoproteota origin.

MAGs reconstructed from deeper peat layers typically had lower numbers of BGCs than those from upper layers, but because genome sizes tended to be smaller in deeper peat, we normalized biosynthetic capacity by genome size. BGC density (BGCs per Mbp) decreased with depth (Fig. 2c, Kruskal–Wallis H(4) = 16.2, *P* = 0.00275, η²H = 0.0216, 95% CI [0.0052, 0.060]), indicating that the lower biosynthetic capacity of MAGs in deeper peat is not an artefact of assembly or MAG quality, but instead reflects a real biological pattern. A similar pattern was observed within the Acidobacteriota, where BGC density also declined with depth, although this decrease was less pronounced than in the full MAG dataset (Fig. 2d, Kruskal–Wallis H(4) = 12.8, *P* = 0.0125, η²H = 0.0695, 95% CI [0.0072, 0.220]). This effect likely reflects the lower biosynthetic capacity of anaerobic taxa, since genomes predicted as anaerobes using *Aerobot*^33^ harboured significantly fewer BGCs per genome than predicted aerobes (two-sided Welch’s *t*-test, *t*(290.17) = 7.62, *P* = 3.56 × 10^−13^, Hedges’ *g* = 0.68, 95% CI [0.49, 0.86]; Supplementary Fig. 4). Accordingly, MAGs obtained from the deepest peat layer we sampled often lacked BGCs entirely, while nearly all MAGs from upper layers encoded at least one (Supplementary Fig. 5).

To test whether this pattern could be explained by differences in MAG or assembly quality, we compared completeness, contamination, contig N50, genome size, GC content and total contigs across depths (Supplementary Fig. 2). MAG quality metrics did not differ, except for slightly higher MAG completeness and higher GC-content in deeper layers. The shift in GC-content is likely due to differences in taxonomic composition between upper and deeper layers.

Several BGC-associated regulator types showed phylum-specific enrichment. Among BGC-associated regulatory genes, Pseudomonadota were enriched in autoinducer-binding regulators (two-sided Fisher’s exact test, OR = 19.95, 95% CI [6.40, 100.52], BH-adjusted *P* = 1.19 × 10^−12^), GntR-family regulator SMCOG1071 (OR = 6.67, 95% CI [2.26, 26.72], BH-adjusted *P* = 2.89 × 10^−4^), and TetR-family regulator SMCOG1057 (OR = 2.46, 95% CI [1.64, 3.76], BH-adjusted *P* = 2.84 × 10^−5^). Conversely, Acidobacteriota were enriched in σ^24^/ECF sigma factors (OR = 3.24, 95% CI [2.36, 4.50], BH-adjusted *P* = 5.03 × 10^−14^), σ^54^-dependent regulators (OR = 2.25, 95% CI [1.63, 3.15], BH-adjusted *P* = 1.74 × 10^−6^), and ArsR-family regulators (OR = 7.12, 95% CI [2.16, 36.94], BH-adjusted *P* = 5.08 × 10^−4^; Supplementary Table 6; Supplementary Fig. 6).

### Diversity of peatland BGCs

To assess biosynthetic novelty, we compared the remaining 8150 peatland BGCs to the BGC Atlas^40^ using BiG-SLiCE v2^41^. This approach supersedes earlier comparisons against the BiG-FAM database^42^, which relied on a BIRCH-based clustering scheme in BiG-SLiCE v1 that is less accurate for shorter BGCs^19,41,43^. Only 233 BGCs (2.9%) clustered into existing gene cluster families in BGC-Atlas^40^, which currently comprises nearly two million BGCs from more than 35,000 metagenomic datasets^40^, whereas the remaining 7917 (97.1%) did not group with any BGC-Atlas^40^ family and thus lacked close relatives in this global reference (Supplementary Fig. 7). We also compared our BGCs to experimentally validated clusters in the MIBiG v4.0^44^ database, which further underscored this novelty, with only 10 BGCs (0.1%) showing close similarity (distance ≤0.4), including matches to Michiganin A, LanII-2D and SRO15-3108 (class II lanthipeptides), microcin B17, phazolicin and kawaguchipeptin A. After removing highly fragmented modular BGCs, a remaining 8140 had distances >0.4 to BGCs in the MIBiG database. Thus, virtually the entire peatland BGC repertoire lies outside both genome-derived and experimentally characterized BGC families, highlighting peatlands as a major reservoir of unexplored biosynthetic diversity.

### Peatland MAGs enriched in BGCs

**Fig. 3:**
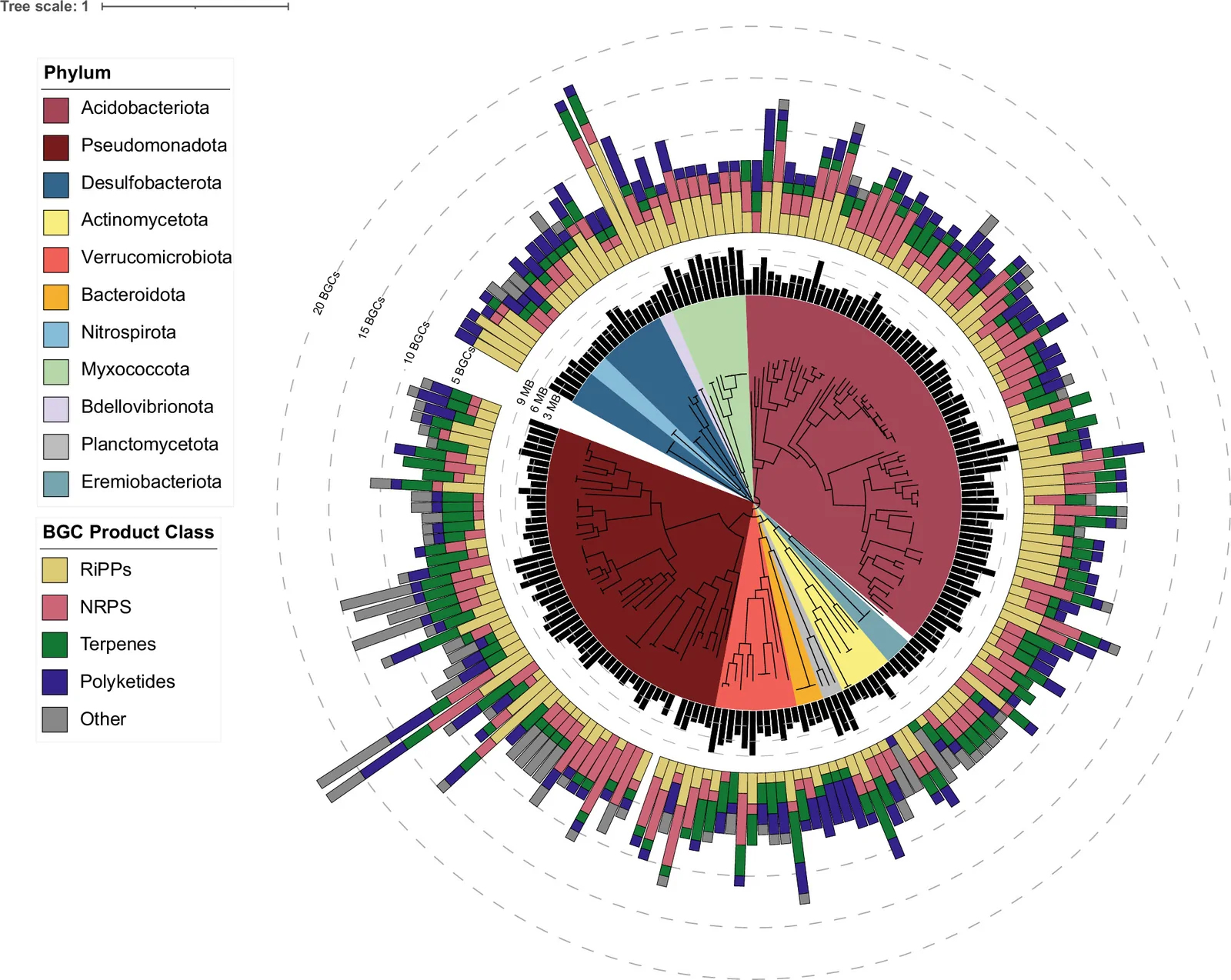
Metagenome-assembled genomes enriched in Biosynthetic Gene Clusters. BGC-rich lineages were identified based on *antiSMASH* predicted BGCs using recovered MAGs as input. Genomes with at least 5 BGCs are shown in the phylogenetic tree. Phyla are indicated as shaded clusters, genome sizes (Mbp) are shown in the inner barplot, outer barplots show the number of respective BiG-SCAPE categorized classes of BGCs. Source data are provided as a Source Data file.

A subset of peatland MAGs stood out as particularly rich in BGCs, with 52 MAGs encoding five or more BGCs (Fig. 3, Supplementary Data 4). The highest counts were from MAGs assigned to Pseudomonadota (formerly Proteobacteria) and Bdellovibrionota. Within Pseudomonadota, two Alphaproteobacteria MAGs each encoded 24 BGCs, including five PKS, five NRPS, and five RiPP clusters. Additionally, 13 other Pseudomonadota (from orders Acetobacterales, Steroidobacterales, Rhizobiales, and Dongiales) carried between 10 and 15 BGCs. We define these MAGs encoding more than 10 BGCs as talented producers.

Two Bdellovibrionota MAGs (family UBA1018) had 17 and 18 BGCs, of which 10 and 12 were RiPPs, respectively. Fourteen Acidobacteriota MAGs had between 10 and 15 BGCs per genome, which were predominantly affiliated with the Terriglobia and Bryobacteriaceae. Additional BGC-rich genomes included three Verrucomicrobiota, two Myxococcota, and one Desulfobacterota_B (all > 10 BGCs). Among MAGs with fewer than 10 BGCs per genome, several showed enrichment in certain BGC classes. Three Syntrophobacteraceae (phylum Desulfobacterota) encoded 5–6 RiPP clusters, while seven Acidobacteriota and six Pseudomonadota MAGs encoded 4–5 and 4–6 NRPS clusters, respectively.

BGCs were also identified in 32 archaeal MAGs, which included a Halobacteriota MAG with four BGCs, including three NRPS clusters, a Thermoproteota and Thermoplasmatota with three BGCs each, and three Halobacteriota had at least two RiPPs. Among these Archaeal MAGs, Thermoproteota also encoded Darobactin, several hydrogen cyanide clusters and RRE-containing clusters, while the Thermoplasmatota also encoded RRE-containing and hydrogen-cyanide clusters.

### Community-wide BGC transcription

**Fig. 4:**
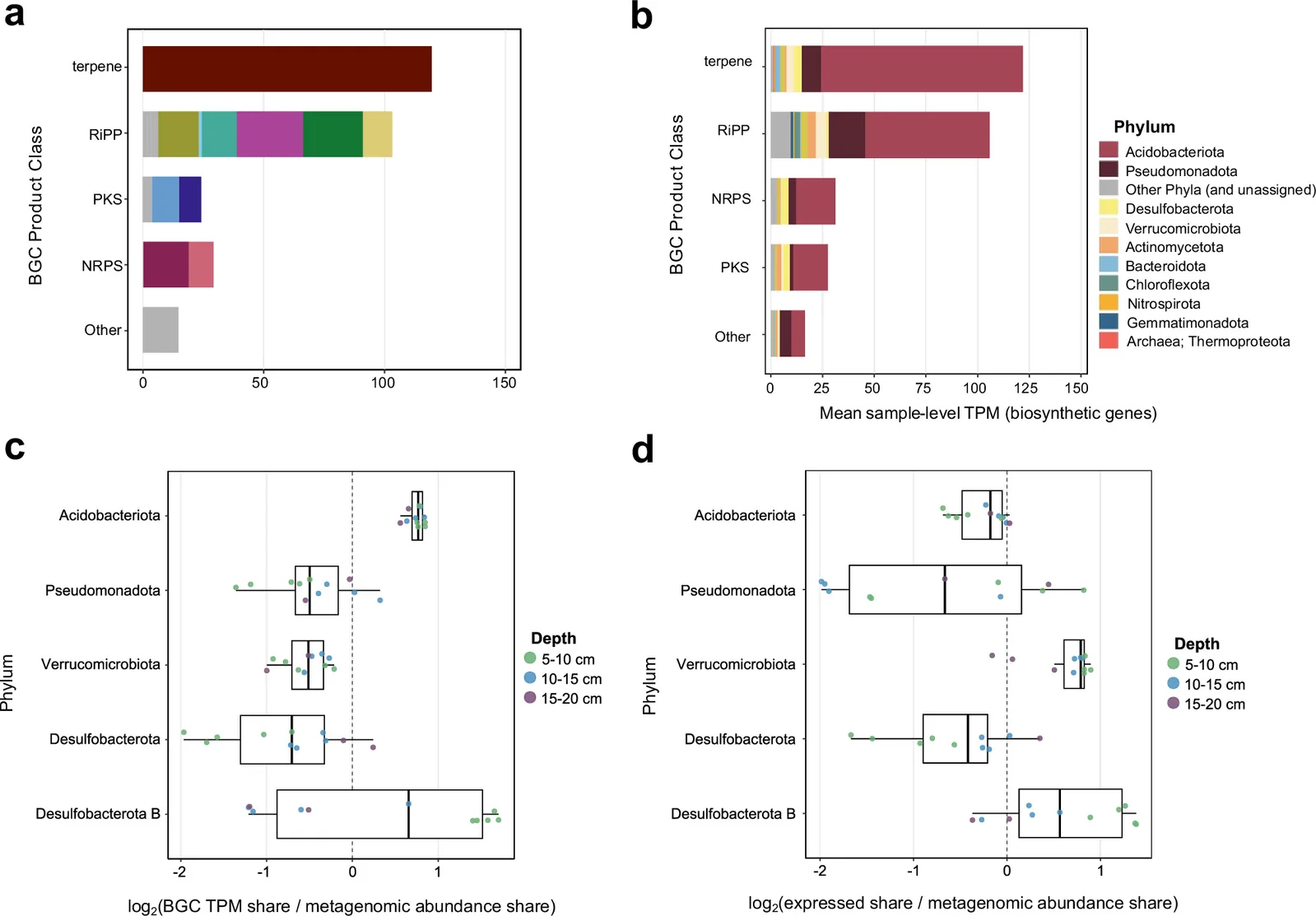
Total expression of core biosynthetic genes from Biosynthetic Gene Clusters. **a** Expression of BGCs shown as the mean transcripts per million (TPM) of biosynthetic core genes by predicted product class for each sample. **b** BGC expression grouped by phylum of the producer also shown as TPM values. **c** Phylum-level enrichment of BGC transcription relative to metagenomic community abundance, calculated from TPM-normalized biosynthetic core-gene expression as log_2_(BGC TPM share/metagenomic abundance share) for each sample, where positive values indicate phyla contributing a larger fraction of total BGC transcripts than expected from their metagenomic relative abundance. **d** Phylum-level enrichment based on number of BGCs expressed, calculated as log_2_(expressed share /metagenomic abundance share) for each sample, where positive values indicate disproportionate BGC transcription relative to phylum-level abundance under a count-based normalization. In (**c**) and (**d**), analyses were based on the same 12 peat samples from three peat depths, and only phyla present in all samples were included. Box plots show the median, 25th and 75th percentiles, and whiskers extending to the most extreme observations within 1.5× the interquartile range; observations beyond the whiskers are shown individually. No inferential statistical tests were performed for (**c**) or (**d**). Source data are provided as a Source Data file.

We first quantified how much BGC transcription occurred in the community overall, and which taxa contributed to it, using contig-level mapping of metatranscriptomic reads so that unbinned community members are included. At contig-level, BGCs were considered as expressed when at least one biosynthetic core gene had TPM > 0.25, corresponding to ~20–40 reads per kilobase. In total, 1120 (13.7%) BGCs were expressed across the dataset. Per sample, terpenes contributed the largest number of expressed BGCs (mean 129, range 113–167), followed by RiPPs (103, 77–137), with smaller contributions from PKS (25, 13–45) and NRPS (29, 16–55). Acidobacteriota expressed the majority of BGCs, with an overall mean of 153 expressed BGCs per sample (range 108–203), corresponding to 68.2% of all expressed BGCs on average (range 59.1–77.1%), followed by Pseudomonadota (39, 13–61). We also summarized the overall investment in BGC expression as the total TPM of core genes. Across depths, and prior to normalization based on taxon-abundance, Acidobacteriota showed the largest share of BGC transcript abundance (mean 200 TPM, 142–343), with smaller but consistent contributions from Pseudomonadota (38 TPM, 14.8–68.8), Desulfobacterota (14.1 TPM, 4.4–47.6) and Verrucomicrobiota (10.2 TPM, 6.2–13.5) (Fig. 4a, b).

Because raw phylum-level BGC expression can reflect differences in taxon abundance, we normalized contig-level BGC transcription by depth-matched metagenomic phylum abundance. Prior to abundance-based normalization of BGC expression, Acidobacteriota contributed the largest raw share of BGC transcription, accounting on average for 68.17% of total BGC TPM and 68.23% of expressed BGCs per sample, while representing 40.6% of the metagenomic community. After abundance normalization, Acidobacteriota remained consistently enriched for BGC expression across all samples, with a median log_2_ enrichment of 0.77 for BGC TPM share and 0.80 for expressed-BGC count share, corresponding to ~1.70- and 1.75-fold enrichment relative to metagenomic abundance, respectively. Desulfobacterota_B also showed positive abundance-normalized enrichment, although with lower raw contribution, whereas Pseudomonadota contributed less BGC transcription than expected from its metagenomic abundance. Thus, the dominance of Acidobacteriota in contig-level BGC transcription reflects not only their high abundance, but also disproportionate BGC expression relative to their community representation (Fig. 4c, d).

### In situ expression of BGC repertoires in individual genomes

Contig-level BGC expression analyses captured total transcriptional output, but cannot resolve how much of any single organism’s biosynthetic repertoire is active. We therefore also performed genome-level expression analyses, where the number of genome-encoded BGCs is known, and where it is possible to determine whether a MAG was active in the community using the expression of single-copy housekeeping genes. MAGs were considered active when the mean TPM values of seven housekeeping genes were higher than 0.5. We defined genome-level biosynthetic potential as the number of BGCs per MAG, from which the proportion of actively expressed BGCs was calculated. Since biosynthetic core genes are essential for metabolite biosynthesis, only those were considered for BGC expression calculations. We observed the expression of accessory BGC genes (linked e.g., to transport, regulation, or unannotated genes) in 801 BGCs in the absence of any reads aligning to biosynthetic core genes.

**Fig. 5:**
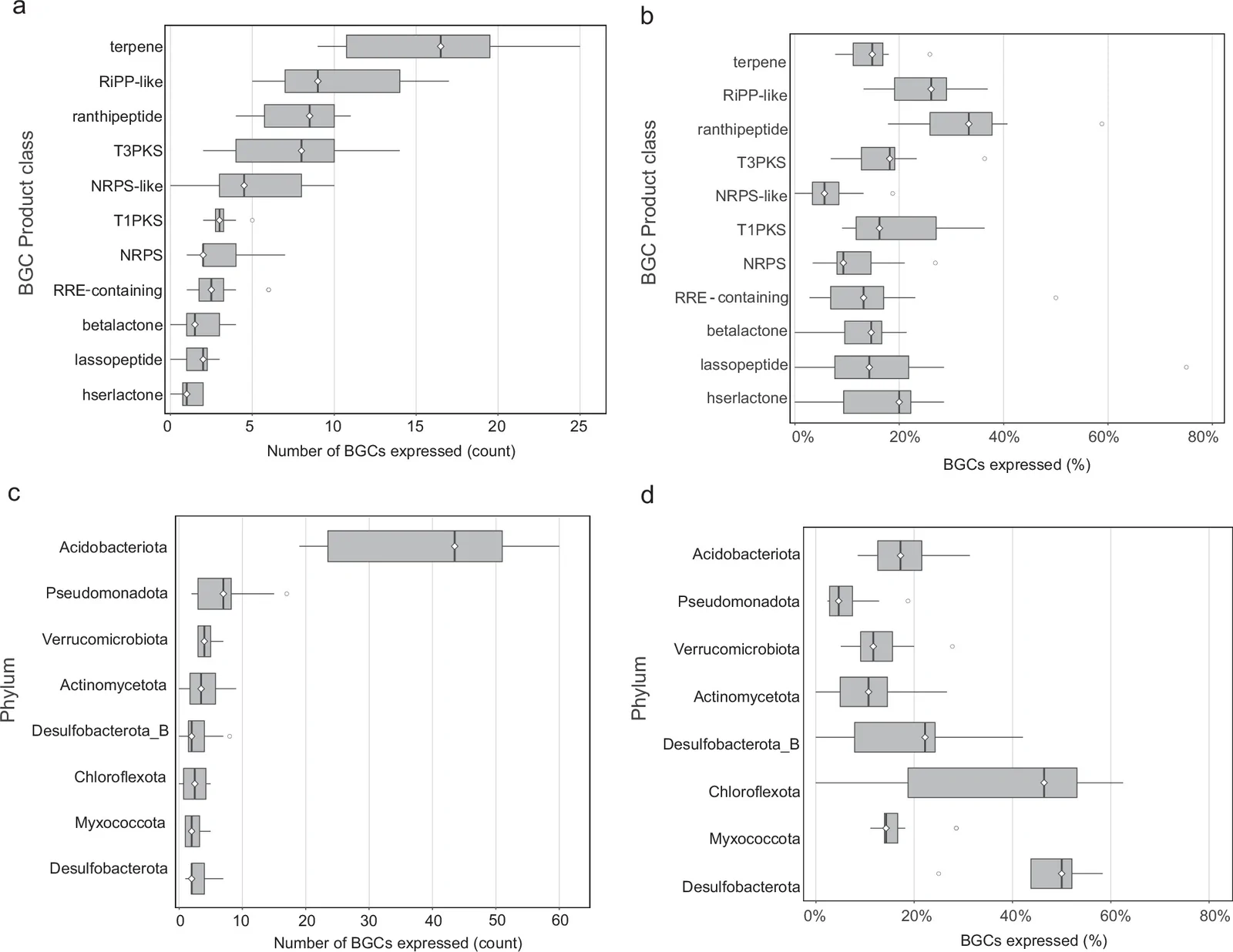
Sample-level expression of biosynthetic gene clusters by product class and phylum. **a** Total number of expressed BGCs per sample, grouped by major BGC product classes as assigned by *antiSMASH*. **b** Proportion of BGCs expressed per sample, grouped by predicted product class. **c** Number of expressed BGCs per sample grouped by the phylum of the predicted producer. **d** Proportion of BGCs present in transcriptionally active MAGs that were considered expressed per sample. In (**a**–**d**), each observation represents one peat sample (*n* = 12 samples collected across three peat depths), depth was not treated as a comparison in these panels, and no technical replicates were treated as independent observations. Box plots show the median, 25th and 75th percentiles, and whiskers extending to the most extreme observations within 1.5× the interquartile range; observations beyond the whiskers are shown individually. No inferential statistical tests were performed; box plots summarize variation among the 12 biological samples. Source data are provided as a Source Data file.

In our MAG dataset, a total of 280 different BGCs were expressed overall, ranging from 41 to 92 BGCs per sample, or 8.8−27% of the biosynthetic potential of the active community (Supplementary Fig. 8). Overall, terpenes accounted for the largest number (67), followed by RiPP-like (43), NRPS-like (29), type III PKS (28), ranthipeptides (28), NRPS (12), RRE-containing clusters (12) and type I PKS (10) (Fig. 5). By proportion, ranthipeptides showed the highest expression across samples (33% on average; 18–59% per sample), followed by RiPP-like (24.4%; 13–37%), type I PKS (20.1%; 9–36%), lassopeptides (18.9%; 0–75%), type III PKS (17.3%; 6.9–36%) and NRPS (11.9%; 3.5–27%) (Fig. 5).

Acidobacteriota MAGs accounted for most expressed BGCs (*n* = 163), followed by Pseudomonadota (40), Desulfobacterota (17), Verrucomicrobiota (15) and Myxococcota (8). Acidobacteriota expressed 19–60 BGCs per sample, representing 8.5–31% (mean 21%) of their encoded clusters, whereas Pseudomonadota expressed 2–17 BGCs per sample, corresponding to 2.4–19% (mean 6.4%). Notably, no transcripts were detected from biosynthetic genes in the two most BGC-rich Pseudomonadota MAGs (24 BGCs each; no006_C20-25_concoct.058 and no007_C25-30_metabat2.335) or the two Bdellovibrionota MAGs with 18 BGCs (no003_B15-20_concoct.178 and no006_C20-25_concoct.049). Verrucomicrobiota MAGs expressed 2-7 BGCs per sample (4–8%).

Because raw phylum-level BGC transcript counts can be inflated in highly abundant taxa, which conflates community abundance with biosynthetic activity, we summarized BGC expression using several metrics in order to normalize BGC activity by abundance and get more realistic BGC expression metrics (Supplementary Fig. 9). Mean TPM per active BGC, as a measure of transcriptional intensity, varied across phyla, with the highest median values in Chloroflexota, Acidobacteriota, Desulfobacterota, Pseudomonadota and Gemmatimonadota (Supplementary Fig. 9A).

Phylum-level BGC expression was normalized using metagenomic relative abundance which showed the highest per-capita BGC activity in Acidobacteriota, Desulfobacterota, Myxococcota, Pseudomonadota and Verrucomicrobiota (Supplementary Fig. 9B). Genome-level BGC expression rates, calculated as the fraction of genome-encoded BGCs expressed per phylum, ranged from 6.1% to 43.7% among phyla with at least 20 encoded BGCs (Fig. 5d). The highest BGC expression rates occurred in Chloroflexota (43.7%), Desulfobacterota (26%) and Acidobacteriota (17.3%), while Pseudomonadota deployed only 6.1% of its BGC-repertoire. Thus, the phyla contributing most to total BGC transcription were not necessarily those deploying the largest fraction of their encoded biosynthetic potential.

Across all genomes, we observed a clear inverse relationship between BGC repertoire size and the fraction of BGCs that were active (two-sided Spearman’s ρ = −0.522, 95% bootstrap CI [−0.634, −0.393], asymptotic *P* = 1.26 × 10^−13^, *n* = 175; Supplementary Fig. 10), indicating that genomes with smaller repertoires tended to express a larger share of their clusters. For Acidobacteriota, this effect was most pronounced below an inflection point of seven BGCs per genome, below which genomes consistently expressed a higher proportion of their biosynthetic capacity than those with larger repertoires. Active Pseudomonadota MAGs, which encoded 3–15 BGCs, expressed no more than five of them in any sample. In contrast, Acidobacteriota encoding 2–13 BGCs routinely expressed a much larger share of their genomic repertoire, including cases of full and near-complete expression. These high expression fractions stand in contrast to the generally low activation rates reported for secondary metabolism in anoxic or energy-limited environments.

To further contextualize differences in the rates of BGC expression, we compared growth- and carbon-processing-associated traits between Acidobacteriota and Pseudomonadota. Predicted doubling times, estimated from codon-usage bias^45^, differed significantly between the two phyla, with lower predicted doubling times in the Pseudomonadota overall (mean 5.77 vs 8.54; mean difference = 2.77, 95% CI [2.03, 3.52]; two-sided Welch’s *t* test, *t*(140.49) = 7.35, *P* = 1.45 × 10⁻¹¹; Supplementary Fig. 11). This pattern was also evident among BGC-rich lineages, where many Pseudomonadota families with ≥5 BGCs had lower mean predicted doubling times than comparably BGC-rich Acidobacteriota families. In contrast, Acidobacteriota encoded significantly more GH-, AA- and PL-class CAZymes associated with complex-carbon degradation than Pseudomonadota (mean 152.6 vs 69.9 annotations per genome; mean difference = 82.7, 95% CI [61.8, 103.7]; two-sided Welch’s *t*-test, *t*(96.01) = 7.84, *P* = 6.22 × 10⁻¹²; Supplementary Fig. 11). They also showed substantially higher expression of carbohydrate-active enzymes, particularly glycoside hydrolases, and across MAGs the fraction of expressed BGCs was strongly positively associated with both glycoside hydrolase expression and total CAZyme expression. Together, these trait differences mirror the contrasting patterns of BGC repertoire size and in situ expression observed across the two phyla.

### MAG-resolved metabolic context of BGC expression

**Fig. 6:**
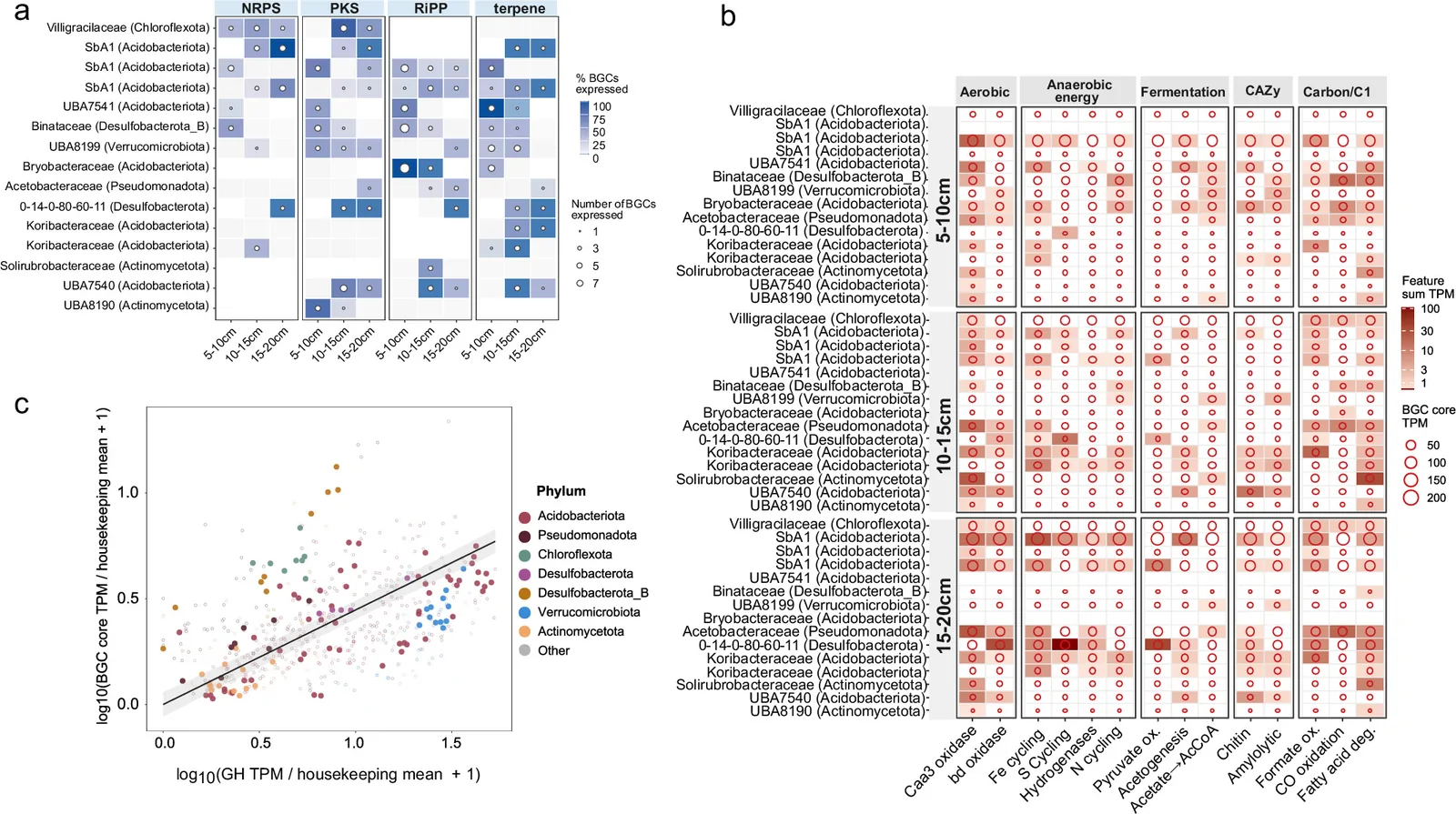
MAG-resolved BGC expression and metabolic context across peatland depths. **a** Percentage of biosynthetic gene clusters (BGCs) expressed per product class for each of 15 focal BGC-encoding metagenome-assembled genomes (MAGs) at three soil depths (5–10, 10–15 and 15–20 cm). Tiles are coloured by the proportion of BGCs in each product class (NRPS, polyketides, terpenes, RiPPs, other) for which core biosynthetic genes were expressed according to the MAG-level biosynthetic core-gene expression criteria defined in Methods and dot size indicates the absolute number of expressed BGCs. **b** Summed expression of metabolic marker genes per functional category for the same MAGs and depths, derived from METABOLIC-G annotations and grouped into aerobic respiration (caa3-type and bd-type cytochrome oxidases), redox/anaerobic energy metabolism (iron cycling, dissimilatory sulfur cycling, hydrogenases, nitrogen cycling), fermentation (pyruvate oxidation, acetogenesis, acetate-to-acetyl-CoA), and carbon processing (chitin degradation, amylolytic enzymes, formate oxidation, CO oxidation, fatty acid degradation). Individual genes grouped into pathways are listed in Supplementary Data 5. Heatmap colour indicates the summed expression of marker genes within each category as log_10_(sum TPM + 1) and cells are blank where the summed expression of all marker genes within a category fell below 0.5 TPM. Red rings on tiles in (**a**) and (**b**) indicate mean BGC core-gene TPM. **c** Glycoside hydrolase (GH) expression, normalised to the mean TPM of single-copy housekeeping genes, versus BGC core-gene expression across all active BGC-encoding MAGs in the dataset coloured by phylum. Filled circles, focal MAGs (*n* = 15); open circles, additional active BGC-encoding MAGs. The black line shows the population-level fixed-effect fit from a linear mixed-effects model with MAG identity and depth as random effects β = 0.447, 95% CI [0.404,0.489], t(547.22) = 20.44, *P* = 1.93 × 10^−69^, the slope did not differ between focal and non-focal MAGs (interaction β = 0.076, CI [−0.025, 0.178], t(545.10) = 1.47, *P* = 0.142). Source data are provided as a Source Data file.

We analysed the relationship between the expression of BGCs and carbohydrate-active enzyme (CAZyme) expression, redox-related metabolic features and KEGG-derived trait modules at the resolution of individual MAGs. We selected 15 BGC-encoding MAGs present across three depth intervals with available metatranscriptomic data resulting in 45 MAG × depth observations (Fig. 6a, b; Supplementary Fig. 12A, B).

BGC expression intensity was closely linked to overall metabolic activity. In separate mixed-effects models, using TPM expression values, normalised to the mean TPM of single-copy housekeeping genes, and with MAG identity as a random effect, BGC core-gene expression was positively associated with glycoside hydrolase expression (*t*(108.89) = 12.37, BH-adjusted *P* = 8.98 × 10^−22^, β = 0.528, 95% CI [0.444, 0.612]), resource-acquisition genes (*t*(107.11) = 11.28, BH-adjusted *P* = 1.63 × 10^−19^, β = 0.622, 95% CI [0.514, 0.731]), anaerobic energy metabolism (*t*(106.61) = 9.90, BH-adjusted *P* = 1.50 × 10^−16^, β = 0.588, 95% CI [0.472, 0.705]), aerobic respiration markers (*t*(104.09) = 8.72, BH-adjusted *P* = 5.87 × 10^−14^, β = 0.481, 95% CI [0.373, 0.589]) and stress-protection markers (*t*(108.25) = 5.96, BH-adjusted *P* = 3.26 × 10^−8^, β = 0.463, 95% CI [0.310, 0.615]; 113 observations from 15 MAGs; Supplementary Data 5). These predictors covaried to differing degrees (pairwise Pearson *r* = 0.071–0.757), and each was positively correlated with total MAG transcriptional activity (*r* = 0.301–0.513). We therefore interpret these associations as evidence that BGC expression generally accompanies metabolically active states rather than indicating a single universal activating physiological state.

Among these metabolic features (Supplementary Data 5), glycoside hydrolase (GH) expression showed the most consistent association with BGC expression intensity. In the 15 focal MAGs, GH expression normalised to the mean TPM of single-copy housekeeping genes in the same MAG was strongly associated with BGC core-gene expression, normalised using the same approach (β = 0.528, 95% CI [0.444, 0.612], *t*(108.89) = 12.37, BH-adjusted *P* = 8.98 × 10^−22^; 113 observations from 15 MAGs). This relationship was also present in BGC-active observations from a much broader set of MAGs (β = 0.447, 95% CI [0.404, 0.489], *t*(547.22) = 20.44, *P* = 1.93 × 10^−69^; 566 observations from 127 MAGs), with no evidence that the slope differed between focal and non-focal MAGs (interaction β = 0.076, 95% CI [−0.025, 0.178], *t*(545.10) = 1.47, *P* = 0.142; Fig. 6c). The association remained strong when raw GH and BGC expression were modelled directly with housekeeping expression included as a covariate (β = 0.525, 95% CI [0.477, 0.573], *t*(558.09) = 21.49, *P* = 4.42 × 10^−75^), indicating that the relationship was not an artefact of shared housekeeping-gene normalization. These results indicate that BGC expression is most consistently associated with carbon-processing activity, particularly GH expression, rather than with a single shared redox or stress state.

We further tested whether this association was also evident in the fraction of genome-encoded BGCs expressed (as opposed to intensity of transcription/TPM above). The proportion of genome-encoded BGCs expressed varied strongly among MAGs, where the mean fraction of genome encoded BGCs that were transcriptionally active ranged from a mean of 0% in an Acetobacteraceae (Pseudomonadota) MAG to 35% in an SbA1 Acidobacteriota MAG, and five MAGs showed BGC expression across all sampled depths (Fig. 6a). Despite this MAG-specific variation, BGC expression usually occurred in a carbon-active context. Across all active MAGs, 97% of BGC-expressing observations also showed GH expression and 99.4% showed CAZyme expression. GH expression was significantly associated with BGC expression in a mixed logistic model accounting for genome and depth effects (*z* = 7.38, *P* = 1.56 × 10^−13^, odds ratio = 57.9, 95% CI [19.7, 170]). Greater GH expression was also associated with expression of a larger fraction of the genome-encoded BGC repertoire after accounting for total MAG transcription, depth and MAG identity (*z* = 5.48, *P* = 4.18 × 10^−8^, odds ratio = 4.78, 95% CI [2.73, 8.35]), whereas total CAZyme expression was nearly ubiquitous and therefore less discriminating as a binary predictor.

Redox-related context was more heterogeneous. Among BGC-active focal MAG-depth observations, BGC expression occurred alongside multiple redox signatures, including microoxic/facultative terminal-oxidase expression, aerobic oxidase expression, anaerobic-function markers, and low or unassigned redox-module expression (Fig. 6b). Thus, no single redox profile was shared across focal MAGs. Instead, individual MAGs combined terminal oxidases, iron cycling, dissimilatory sulfur cycling, hydrogenases, nitrogen cycling and fermentation-related markers in different ways across depths.

Overall, the MAG-resolved analysis shows that BGC expression in peatland MAGs is not governed by a single physiological state. Rather, BGC expression is MAG-specific and occurs across a mosaic of carbon-processing, resource-acquisition, stress-protection and redox contexts. Across this heterogeneity, the most consistent shared signal was carbohydrate-active enzyme expression, particularly GH expression, indicating that BGC expression is most closely associated with carbon-active MAGs while remaining lineage- and depth-specific.

## Discussion

The vast biosynthetic potential of uncultivated microbes is now well established^40,46^, yet how this capacity is realized in natural environments remains poorly resolved. Based on only seven metagenomes, we could show that the studied peatland microbiomes harbour nearly 10,000 BGCs, comparable in scale to global surveys, yet only 8–27% were transcribed in situ, indicating that environmental and physiological constraints strongly govern secondary metabolism. This selective activation shows that genomic capacity alone poorly predicts realized chemistry and highlights metabolic strategy, carbon-processing activity, and taxon-specific life-history as key determinants of BGC expression. Peatlands thus offer a powerful system for examining how ecological context shapes biosynthetic functions.

Peatland BGCs exhibited an exceptionally high degree of novelty relative to existing genomic and natural product reference databases, reflecting contributions from both classically recognized producers and phylogenetically understudied lineages. These taxa, abundant but historically understudied due to cultivation challenges, harbor some of the richest repertoires of BGCs identified to date, including NRPS, PKS, and RiPP clusters typically associated with well-known producers such as Actinobacteria or Pseudomonadota. Common terrestrial taxa such as Acidobacteriota and Verrucomicrobiota, only recently explored for secondary metabolism^18,20,24^, were major contributors in our dataset, with Acidobacteriota spanning multiple families and metabolic strategies^47,48^ emerging as especially prolific and transcriptionally active producers. Their prominence suggests that broad surveys of Acidobacterial genomes and expression dynamics may reveal substantial, currently overlooked chemical diversity promising for bioprospecting.

We also detected numerous BGCs in anaerobic lineages, such as Chloroflexota, Desulfobacterota, and Planctomycetota, groups recognized as sources of novel biosynthetic capacity in mangrove sediments and anoxic marine systems^49–51^. Their presence underscores that novelty arises not only from classical soil phyla but also from anaerobes adapted to the groundwater-fed, redox-fluctuating conditions of the slightly acidic Schlöppnerbrunnen fen, conditions that support methanogenesis as well as Fe- and sulfate reduction^28,52,53^. Notably, Desulfobacterota, the key sulfate reducers in this fen^54^, both encoded and actively expressed BGCs, demonstrating that even oxygen-sensitive and energetically constrained guilds contribute to the chemical complexity of peatland microbiomes.

Archaeal lineages, such as Thermoproteota and Halobacteriota, also encoded diverse BGCs, including darobactin-like RiPP clusters, a group reported to have antibiotic activity against Gram-negative bacteria^55^. The presence of genes associated with hydrogen cyanide biosynthesis in Archaea, known to inhibit cytochrome c oxidases and nitrogenases^56,57^, alongside additional NRPS- and RiPP-like BGCs further indicates that archaeal secondary metabolites may confer competitive or defensive advantages in redox-dynamic habitats.

The coexistence of aerobic decomposers, facultative anaerobes, and strict anaerobes in peatlands creates an unusually heterogeneous biosynthetic landscape. Classical soil taxa and redox specialists jointly expand the known boundaries of secondary metabolism, making peatlands a unique reservoir of chemical novelty shaped by sharp environmental gradients and niche diversification.

In situ BGC expression was dominated by Acidobacteriota, even though the largest biosynthetic repertoires occurred in Alphaproteobacteria and Bdellovibrionota talented producers. This decoupling between genomic potential and transcriptional activity reflects fundamental contrasts in life-history strategy. Predicted doubling times and complex carbon utilization potential was lower in the Pseudomonadota, which are often characterized by genomes enriched in nutrient uptake and transport systems and with high metabolic turnover, motility, and rapid responses to nutrient availability^45,58,59^. These traits come at substantial energetic cost and are typically favoured in resource-rich, oxygenated environments. Thus, their extensive BGC repertoires may therefore represent latent genomic capacity that is deployed only under conditions that meet the regulatory and energetic requirements for activation, conditions that are uncommon or short-lived in peatlands.

BGC-expressing Acidobacteriota encoded more CAZymes per genome and showed significantly higher in situ CAZyme expression, especially glycoside hydrolases. Across a subset of 15 highly active BGC-expression MAGs spanning seven phyla, BGC core-gene expression was strongly associated with GH and total CAZyme expression. Although Acidobacteriota MAGs encode fewer BGCs than Pseudomonadota, they consistently expressed a much larger fraction of their biosynthetic repertoire, indicating that secondary metabolism is more tightly integrated into their ecological strategy, and continued activity as carbon degraders. Pseudomonadota, which had significantly lower predicted doubling times and smaller CAZyme repertoires expressed comparatively fewer BGCs despite having much larger genomic BGC repertoires. This pattern suggests that secondary metabolism in peatland Acidobacteriota is coupled to active carbohydrate processing and is more tightly integrated into their ecological strategy than in faster-growing taxa with larger but largely silent BGC repertoires.

These ecological differences were evident in regulatory repertoires. Pseudomonadota BGCs were enriched in autoinducer-binding regulators and transcription factors, such as GntR and TetR, which are often associated with quorum sensing, competition and motility^60,61^. Such systems require high cell densities or nutrient cues. In contrast, Acidobacteriota were enriched in ArsR-family regulators and σ^24^ (ECF)- and σ^54^-dependent systems linked to environmental sensing and stress-responsive control^62,63^, which allows coupling between environmental stress and BGC activation. These patterns suggest that BGC expression reflects the distinct metabolic strategies of different peatland microbial lineages. Acidobacteriota exhibited a clear coordination between BGC expression and active carbohydrate metabolism, whereas Pseudomonadota encoded larger biosynthetic repertoires that were weakly deployed. This suggests that while some taxa integrate secondary metabolism with resource acquisition, which includes complex carbon degradation, others maintain latent biosynthetic capacity not activated under these conditions.

Despite this selective activation, only a minority of genome-encoded BGCs were expressed across samples. At the genome level, BGC transcription was sparse: the median active MAG expressed only a single BGC, and 52% of active MAGs expressed exactly one. Only a small subset expressed three or more clusters (maximum of seven), and these multi-BGC-active genomes were strongly enriched in Acidobacteriota. This within-genome sparsity mirrors observations from cultured model organisms such as Streptomyces, where up to 90% of BGCs remain silent under standard laboratory conditions^64^. Such low activation rates reflect the high metabolic cost of secondary metabolite biosynthesis, which diverts precursors from primary metabolism and relies on multilayered regulatory systems integrating nutrient status, stress responses, and community-level cues^65–68^. In peatlands, these constraints appear to selectively favour Acidobacterial BGC activation during periods of active carbon processing and resource acquisition, while rendering the larger but more energy-demanding Pseudomonadota repertoires largely dormant.

At the MAG-resolved level, BGC expression occurred across heterogeneous redox and metabolic contexts, including in aerobic, microoxic, and anaerobic-associated conditions with no single physiological state shared across active taxa. All measured functional modules co-varied positively with BGC expression intensity, consistent with BGC transcription scaling broadly with overall MAG metabolic activity. Among the functional modules tested, glycoside hydrolase expression showed a particularly strong and consistent positive association with BGC expression, although positive associations were also observed for resource acquisition, aerobic and anaerobic energy metabolism, and stress protection. These results are consistent with BGC expression being most tightly coupled to active carbohydrate processing rather than to energetic or stress cues specifically. These patterns indicate that BGC expression in peatland MAGs reflects metabolically active, carbon-processing states rather than constitutive expression or a specific stress response.

BGC expression was detected across all peat depths, including in deeper, more anoxic layers. This suggests that secondary metabolism persists even where biosynthetic capacity is reduced and energy availability is limited. In deeper peat layers, BGCs expression occurred across a range of co-expressed redox contexts, including anaerobic and microoxic associated functions, suggesting that secondary metabolism persists through diverse metabolic modes rather than being confined to high-energy oxic environments. Functional shifts toward redox-active or protective secondary metabolites have been reported in anoxic marine sediments and cold seeps^49,50^. The ability of Acidobacteriota to express BGCs in both oxic surface layers and deeper anoxic zones underscores their ecological breadth, enabling them to compete with fast-growing aerobic decomposers as well as slow-growing anaerobic specialists. Sustained BGC activity across depths thus points to a broader ecological function in stress tolerance and metabolic stability under fluctuating oxygen and nutrient conditions.

Our study shows that cataloguing BGC diversity alone is insufficient to infer ecological function. Although peatland microbes encode nearly 10,000 BGCs across a broad phylogenetic range, only a small fraction is expressed in situ, and expression is shaped more by physiological state and environmental conditions than by genomic capacity. Acidobacteriota were the dominant contributors to BGC transcription, even in deeper, energy-limited peat layers, indicating that secondary metabolism is a consistent feature of their carbon processing ecology across the redox gradient. Our findings reveal that heterogeneous, redox-structured environments harbor extensive biosynthetic novelty, but only select conditions trigger its activation. BGCs are everywhere, but the environment selects.

## Methods

### Site description and sampling

Peat soil cores were collected from the Schlöppnerbrunnen fen, located in the northern Fichtelgebirge, Northern Bavaria, Germany (50°07′55″N, 11°52′52″E). The fen covers ~0.8 ha and is characterized by slightly acidic soils on granite bedrock. Vegetation is dominated by *Carex canescens*, *Carex rostrata*, *Juncus effusus*, *Molinia caerulea*, and *Eriophorum vaginatum*. Peat accumulation varies between 30 and 70 cm depth^52^. The Schlöppnerbrunnen fen is fed by anoxic, Fe(II)-rich groundwater leading to an enrichment of total iron in the upper peat layers. Groundwater levels fluctuate between 10 and 30 cm below the surface, influencing mean oxygen penetration depths, which was previously reported to be up to around 16 cm deep^69^. Across depths of 0–30 cm, the fen has a pH of ~4.7, total iron content of 105–170 µmol.g^−1^ ^28,52^ and sulfate content of 20–240 µM^70^. The Schlöppnerbrunnen fen has been the focus of extensive biogeochemical and microbial research^27,28,53,70,71^.

For this study, we analysed data from two parallel peat cores (B, and C) collected in July 2020 using a soil corer. Cores were sectioned in the field at 5 cm intervals down to 30 cm depth, and each segment was homogenized. Samples were flash frozen, transported on dry ice, and stored at −80 °C until DNA and RNA extraction. Due to the very dry conditions in the field, peat from the upper 0–5 cm depth was not included in the extractions. Despite multiple attempts, RNA could not be recovered from the deeper 20–30 cm layers, likely due to inhibitory compounds and high iron content.

### Nucleic acid extraction and sequencing

Total RNA was extracted using the RNeasy Powersoil Total RNA Kit (Qiagen, Hilden, Germany) following the manufacturer’s instructions. Potentially present genomic DNA was digested with TURBO^Tm^ DNase (ThermoFisher Scientific, Darmstadt, Germany) before being purified using the RNA Clean and Concentrator-5 kit (ZymoResearch, Freiburg, Germany). Purified RNA was quantified through fluorometry, using a Qubit® 3 fluorometer, and RNA HS assay reagents (ThermoFisher Scientific). Purity was assessed using spectrophotometry with a NanoDrop® microvolume spectrophotometer (ThermoFisher). RNA integrity was checked using agarose gel electrophoresis. RNA samples were subjected to Illumina sequencing library preparation using the NEBNext Ultra II Directional RNA Library Prep kit (New England Biolabs, Frankfurt am Main, Germany), aiming for an insert size of ~200 bp. Finalized libraries were quantified through fluorometry, and their size distribution was checked using a Bioanalyzer® instrument (Agilent Technologies, Waldbronn, Germany) and the DNA 7500 kit. Sequencing was carried out at the sequencing core facility of the Leibniz Institute on Aging - Fritz Lipmann Institute on an Illumina NovaSeq device using an S2 flowcell in paired-end mode (2 ×150 bp).

### Sequence data processing

Metagenome reads were trimmed using *TrimGalore* (v0.6.6)^72^ with a specified --length of 75 and --quality of 28 to remove low quality end bases and filter out sequences shorter than 75 bp. Forward and reverse reads were merged using *Flash* (v1.2.11)^73^ with a --max-overlap of 151 bp. Metagenome sequences were assembled using *SPAdes*^74^ (v3.15.3) in metagenome mode with kmer sizes 21, 33, 55, 77, 99, 111, and 127. Metagenome coverage/redundancy was estimated using Nonpareil v3.5.5^75^. Assemblies >2000 bp were binned using *MetaBat2* (v2.15)^76,77^, *Concoct* (v1.1.0)^78^, and *Maxbin* (v2.2.5)^79^. Bins were then evaluated with *DAS Tool* (v1.1.2)^80^ to select the best non-overlapping bins. A selection of 573 bins from seven peat samples, had completeness >70% and contamination <10% which were used for further analyses (Supplementary Table 3). Bin quality, percent of binned population, and estimated percent of community were assessed using *CheckM2* (v1.0.2)^81^.

### Annotation

Gene calling was done using *Prodigal* (v2.6.3), as implemented in *Anvi’o* (v8)^82,83^, and then annotated using *kofams*^84^, *COG20* and *CAZy* (*dbCAN2*)^85^ databases using the commands *anvi-run-kegg-kofams*, *anvi-run-ncbi-cogs* and *anvi-run-cazymes* respectively. Additional annotations were done for MAGs using *METABOLIC* (v4.0), using the *METABOLIC-G.pl* script^86^. Gene calls were exported from *Anvi’o* contig databases using the *anvi-get-sequences-for-gene-calls* command with the *– --export-gff3* setting. Contig-level taxonomy was determined using *MMseqs2* (v15-6f452) *MMseqs2*^87^, using *GTDB*  (release 220)^88^. MAG taxonomic annotation was performed using *GTDB-tk*^ (v2.4.0)89^, using *GTDB* Release 220.

Oxygen-utilization phenotypes were predicted from MAGs using *Aerobot*^33^ and *Oxyphen*^34^. *Aerobot* classifies genomes as aerobic, anaerobic or facultative using machine-learning models trained on amino-acid composition^33^. *Oxyphen* predicts oxygen phenotype from proteomes based on the number and identity of encoded O₂-utilizing enzyme classes, which distinguish aerobic from anaerobic organisms^34^. Growth rates were predicted for MAGs from genome-wide codon usage bias using *gRodon2*^45^, run in full mode on *Prodigal* predicted coding sequences with ribosomal proteins identified from KEGG annotations used as the highly expressed gene set.

### BGC identification

Biosynthetic gene clusters (BGCs) were identified from metagenome contigs longer than 5 kb using *antiSMASH*^35^ (v7.0.1) with the settings *–fullhmmer –pfam2go –cc-mibig –cb-subclusters –rre –smcog-trees –asb –cb-knownclusters*.

To assess the potential inflation of raw BGC counts caused by fragmented short-read assemblies, we performed a sensitivity analysis on the position of *antiSMASH*-annotated biosynthetic core genes relative to contig ends. Absolute gene positions were reconstructed from *antiSMASH* region coordinates and parent contig lengths. A BGC was considered to contain an anchored biosynthetic core gene if the gene boundaries of at least one biosynthetic core gene was located more than 5 kb from the nearest contig end. We then defined weak fragments conservatively as contig-edge NRPS, PKS or NRPS-PKS hybrid BGCs lacking any anchored biosynthetic core gene. These weak fragments were used in sensitivity analyses of BGC counts and GCF richness, and were not interpreted as false positives, but as the subset most likely to contribute to overestimation of modular BGC counts in short-read assemblies.

The fragmentation-filtered BGC dataset was further clustered into biosynthetic gene cluster families (GCFs) as a means to dereplicate them using *BiG-SCAPE* (v1.1.9)^21^ with settings *--include_singletons --mibig --mode auto*and 0.3 similarity threshold. Clustering in *BiG-SCAPE* is done through the calculation of pairwise distances between BGCs using a weighted combination of the Jaccard, Adjacency Index, and Domain Sequence Similarity indices. Furthermore, *BiG-SCAPE* classifies BGCs into terpenes, NRPS, RiPPs, PKSother, PKS type I, NRP-PKS hybrids, and others, and these groups are hereafter referred to as *BiG-SCAPE* classes. BGC abundance in metagenomes was calculated by alignment of previously quality-controlled metagenome reads against the seven metagenome assemblies using *bowtie2*^90^ (v2.4.5). BGC novelty was assessed by comparing GCFs against the BGC-Atlas^40^ *BiG-SLiCE* 2.0^41^ using the ‘query’ function. BGCs from MIBiG 4.0^44^ were first clustered using the clustering function with a distance threshold of 0.4 followed by comparison with BGCs from this study using the *‘query’* function. BGC-Atlas comprises more than two million BGCs from various metagenomic sources, while the MIBiG database contains all experimentally validated BGCs.

### Metatranscriptomics

Raw metatranscriptome reads were quality filtered and sequencing adaptors removed using *fastp*^91^ (v0.23.4) with default settings, followed by rRNA filtering using *Ribodetector*^92^ (v0.3.1), using default settings. For calculation of total BGC transcription, ribosomal RNA-depleted QC-reads were aligned to whole assemblies using *bowtie2* .

For MAG-level analyses, ribosomal RNA-depleted, quality-filtered reads were aligned to a dereplicated set of MAGs (*n* = 369; 98% average nucleotide identity, *dRep*^93^ (v2.6.2), --S_ani 0.98). Read mapping was performed with *Bbowtie2* using default settings, and SAM files were converted to BAM with *samtools*^94^ (v1.7). Aligned BAM files were then filtered with *reformat.sh* from *BBTools*^95^ (v39.01) using *pairedonly=t*, *primaryonly=t* and *idfilter=0.95*, and converted to gene counts with *featureCounts* (v2.0.6) ^96^ using *-s 0 -t CDS -p --countReadPairs*. In total, 131 million read pairs mapped to MAGs (4.5–20.8 million per sample). Counts <5 per sample and <20 across all samples were removed, and remaining counts were normalized to transcripts per million (TPM) in R.

BGC expression was assessed using different criteria in the contig-level and MAG-level analyses. In the contig-level dataset, a BGC was classified as expressed if at least one biosynthetic core gene had TPM > 0.25 (~20–40 reads per kilobase). For MAG-level analyses, expression was evaluated only in active MAGs, defined as genomes with a mean TPM > 0.5 across seven single-copy housekeeping genes: *rpoB* (K03043), *rpoC* (K03046), *gyrA* (K02470), *tuf* (K02358), *infB* (K02519), *atpD* (K02112), and *sigA* (K03086). BGCs were considered expressed when core biosynthetic genes, as identified by *antiSMASH* ^35^, met at least one of the following criteria: (i) mean biosynthetic core-gene TPM ≥ 75% of the mean TPM of the housekeeping genes, (ii) any core gene with TPM greater than the genome-wide housekeeping-gene mean, or (iii) biosynthetic core gene TPM > 0.8, corresponding approximately to 20–30 reads per kilobase. Multi-mapping of metatranscriptomic reads was minimized by dereplicating MAGs at 98% ANI prior to mapping.

Across samples, the mean number of active MAGs was 82 (range 45–119 per sample) from the 349 MAGs used for BGC expression mapping. These active MAGs contained 282–680 BGCs per sample, corresponding to 969 distinct BGCs across all samples, which we refer to as the total biosynthetic potential. Of these, 726 BGCs were detected as expressed at least once, representing 280 unique BGCs.

### Data analyses

All statistical and associated data analyses were performed in R version 4.3.0^97^, using the packages *tidyverse (v2.0.0)*^98^ *lme4 (v1.1.34)*^99^, and *lmerTest (v3.1.3)*^100^ unless stated otherwise. The packages *dplyr* (v1.1.2)^101^, *forcats* (v1.0.0)^102^ and *stringr* (v1.5.0)^103^ were used for processing large data tables, and *ggplot2* (v3.5.2)^104^ was used to generate all figures, except for Fig. 3 which was created using *iTol* (v6)^105^.

To test whether MAG quality or assembly characteristics varied with depth, we compared completeness, contamination, contig N50, GC content, genome size and total contig number across depth groups using Kruskal–Wallis tests followed by Dunn’s post hoc tests with Benjamini–Hochberg correction. Because BGC repertoire size was positively associated with genome size, depth-related differences in biosynthetic capacity were evaluated using both raw BGC counts per MAG and BGC density normalized by genome size (BGCs per Mbp)

Differences in biosynthetic capacity between groups of MAGs were assessed using two-sided Welch’s *t*-tests using the *t.test* function in base R^97^ (v4.3.0). Genomes predicted as anaerobes and aerobes were compared for the number of BGCs per genome. The relationship between BGC repertoire size and the fraction of BGCs expressed per MAG was quantified using Spearman’s rank correlation, using the *cor.test* function in base R.

Enrichment of BGC-associated regulator types between Acidobacteriota and Pseudomonadota was assessed using two-sided Fisher’s exact tests on SMCOG-annotated regulator counts extracted from *antiSMASH* BGC annotations. Odds ratios and their 95% confidence intervals were used as effect-size estimates, and *P* values were adjusted for multiple comparisons using the Benjamini–Hochberg procedure using the *fisher.test* and *p.adjust* commands in base R.

We selected 15 highly active, focal Metagenome-Assembled Genomes (MAGs) spanning the Acidobacteriota, Pseudomonadota, Chloroflexota, Desulfobacterota, Desulfobacterota_B, Verrucomicrobiota and Actinomycetota to examine the metabolic context of BGC expression. MAGs were included if they showed reliable housekeeping-gene activity and met one of three BGC criteria: (i) ranked in the top 25 MAGs for total BGC expression, (ii) encoded ≥5 BGCs and expressed ≥25% of them, or (iii) expressed BGCs across multiple depth layers.

To account for baseline transcriptional activity, we normalized BGC and predictor TPMs by dividing them by the mean single-copy housekeeping gene TPM for that genome/sample. Samples with mean housekeeping TPM < 0.5 were excluded. For visualizations, functional features were summed per category, filtered to remove negligible activity (<0.5 TPM), and log-transformed as log_10_(normalized expression +1).

We tested associations between BGC expression and functional predictors (like GH expression) using linear mixed-effects models, treating MAG identity and depth as random intercepts. To avoid collinearity, predictors were modelled separately. We used mixed logistic models and Fisher’s exact tests to determine if BGC expression coincided with CAZyme expression, applying Benjamini–Hochberg corrections for multiple testing.

### Reporting summary

Further information on research design is available in the Nature Portfolio Reporting Summary linked to this article.

## Supplementary information

Supplementary information

Description Of Additional Supplementary Files

Dataset 1

Dataset 2

Dataset 3

Dataset 4

Dataset 5

Reporting Summary

Transparent Peer Review file

## Source data

Source Data

## Data Availability

Raw sequencing data generated in this study is available in the NCBI Sequence Read Archive under BioProject PRJNA1374247. Metagenome assembled genomes (MAGs) and Biosynthetic gene clusters generated in this study have been deposited in Zenodo under accession https://doi.org/10.5281/zenodo.17812908. Source data are provided with this paper.

## Code availability

Code used for data analysis and to generate figures, along with additional data related to this manuscript are available at Zenodo (https://doi.org/10.5281/zenodo.17812908).
