# Supplementary material for: High diversity but limited expression of biosynthetic gene clusters in a peatland microbial community
Source: Nat Commun. 2026 Oct 6;17:10529. doi: 10.1038/s41467-026-78121-8 (PMC13642348; doi:10.1038/s41467-026-78121-8)
Supplement: Supplementary file 1 [file 41467_2026_78121_MOESM1_ESM.pdf]

High diversity but limited expression of biosynthetic gene clusters in a peatland microbial community

Zander Rainier Human[1], Rene Hoover[2,3], Clara Chan[2,3], Kirsten Küsel[1,4,5,#], Carl-Eric Wegner[1,6,#]

[1]Institute of Biodiversity, Ecology & Evolution, Aquatic Geomicrobiology, Friedrich Schiller University Jena, Dornburger Str. 159, 07743 Jena, Germany

[2]Department of Earth Sciences, University of Delaware, 590 Avenue 1743, Newark, DE 19713, United States of America

[3]Microbiology Graduate program, University of Delaware, Newark, DE 19713, United States of America

[4]Cluster of Excellence Balance of the Microverse, Friedrich Schiller University Jena, Jena, Germany

[5]German Center for Integrative Biodiversity Research (iDiv) Halle-Jena-Leipzig, Puschstraße 4, 04103 Leipzig, Germany

[6]Department of Inorganic Chemistry, Bioinorganic Chemistry, Heinrich Heine University Düsseldorf, Universitätsstr. 1, 40225 Düsseldorf, Germany

[#]corresponding authors: Kirsten Küsel | kirsten.kuesel@uni-jena.de

Carl-Eric Wegner | carl-eric.wegner@hhu.de

Supplementary Figures

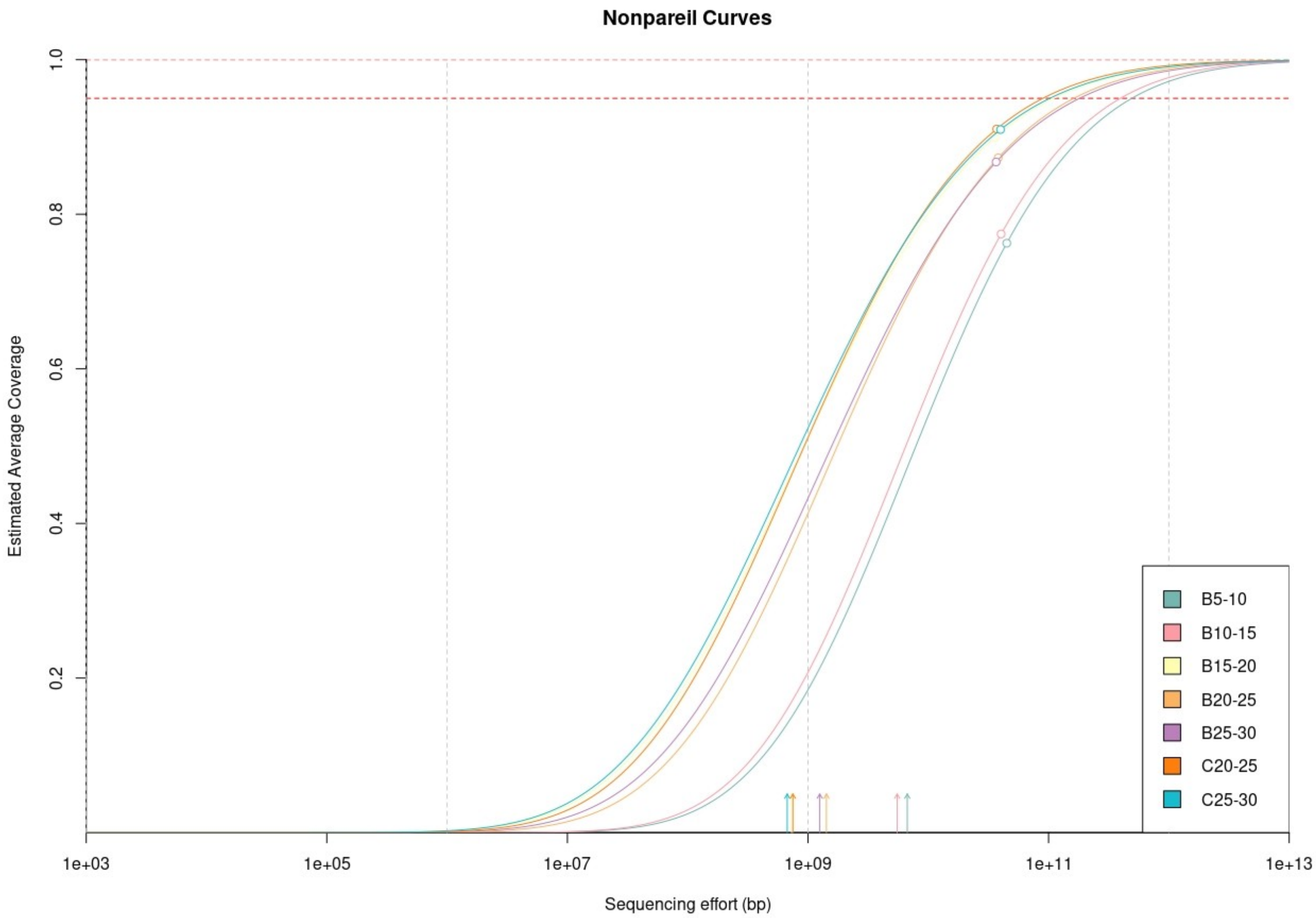

Supplementary Figure 1 | Sequencing coverage and complexity of peat metagenomes.

Nonpareil3[1] estimates of read redundancy (κ), coverage (C), and diversity for each metagenome. Points indicate observed redundancy, fitted curves yield coverage estimates. Source data are provided as a Source Data file.

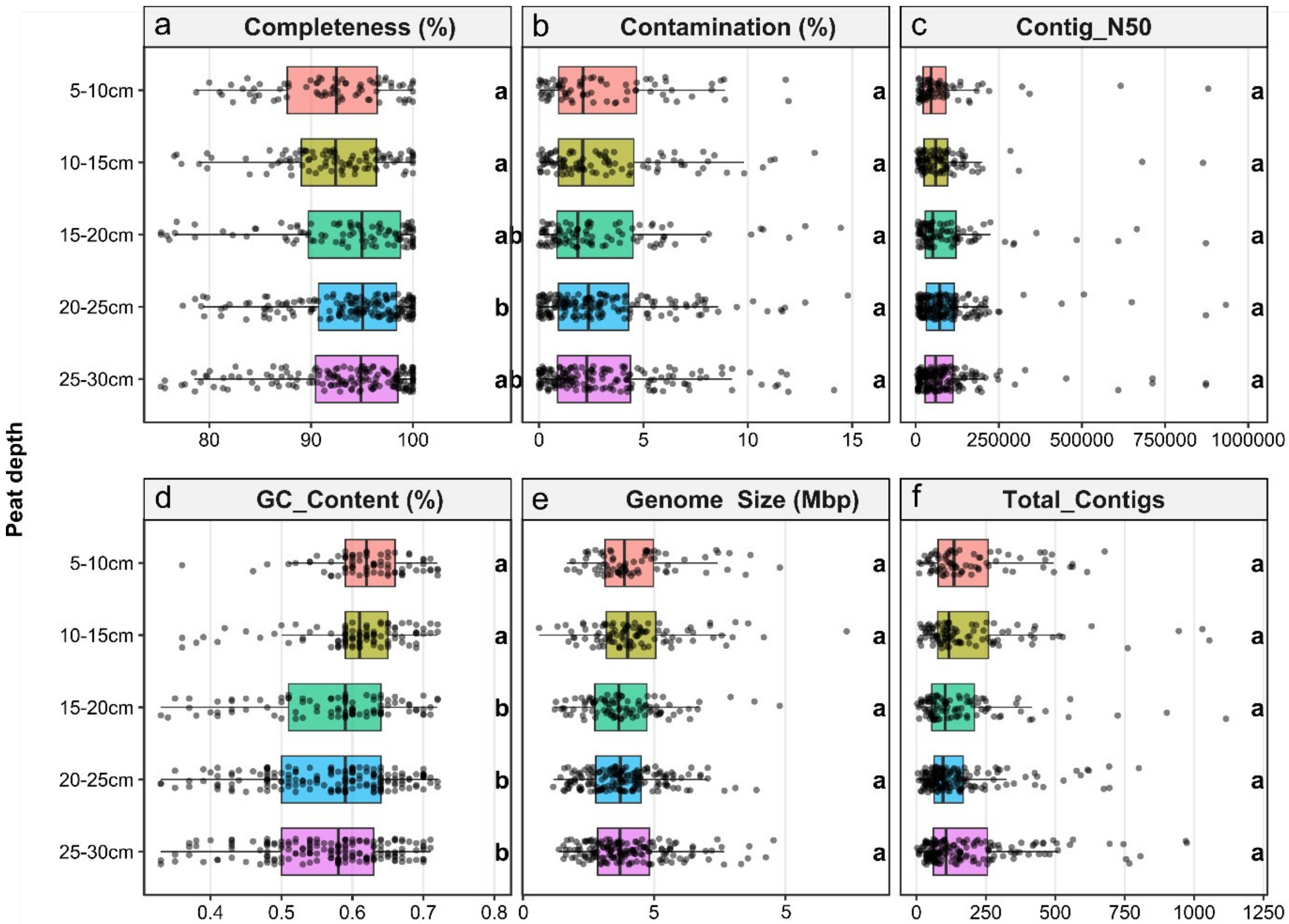

Supplementary Figure 2 | MAG quality and assembly statistics across peat depths. Boxplots show the distributions of (a) completeness, (b) contamination, (c) contig N50, (d) GC content, (e) genome size and (f) total contig number for metagenome-assembled genomes (MAGs) recovered across peat depths. Points represent individual MAGs. Boxes indicate the median and interquartile range (IQR), and whiskers extend to the most extreme values within 1.5 × IQR. Sample sizes were n = 74, 96, 95, 166 and 164 MAGs for the 5–10, 10–15, 15–20, 20–25 and 25–30 cm depth intervals, respectively. Differences among depths were assessed independently for each metric using two-sided Kruskal–Wallis tests followed by Dunn's pairwise tests with Benjamini–Hochberg correction for multiple comparisons. Significant overall differences were detected for completeness (Kruskal–Wallis H = 13.3, df = 4, P = 0.00985) and GC content (H = 32.1,

df = 4, $P = 1.81 \times 10^{-6}$), whereas contamination ($P = 0.995$), contig N50 ($P = 0.306$), genome size ($P = 0.156$) and total contig number ($P = 0.0517$) did not differ significantly among depths. Different letters indicate significant Dunn pairwise comparisons at Benjamini–Hochberg-adjusted $P < 0.05$. Exact pairwise P values are provided in the Source Data. Source data are provided as a Source Data file.

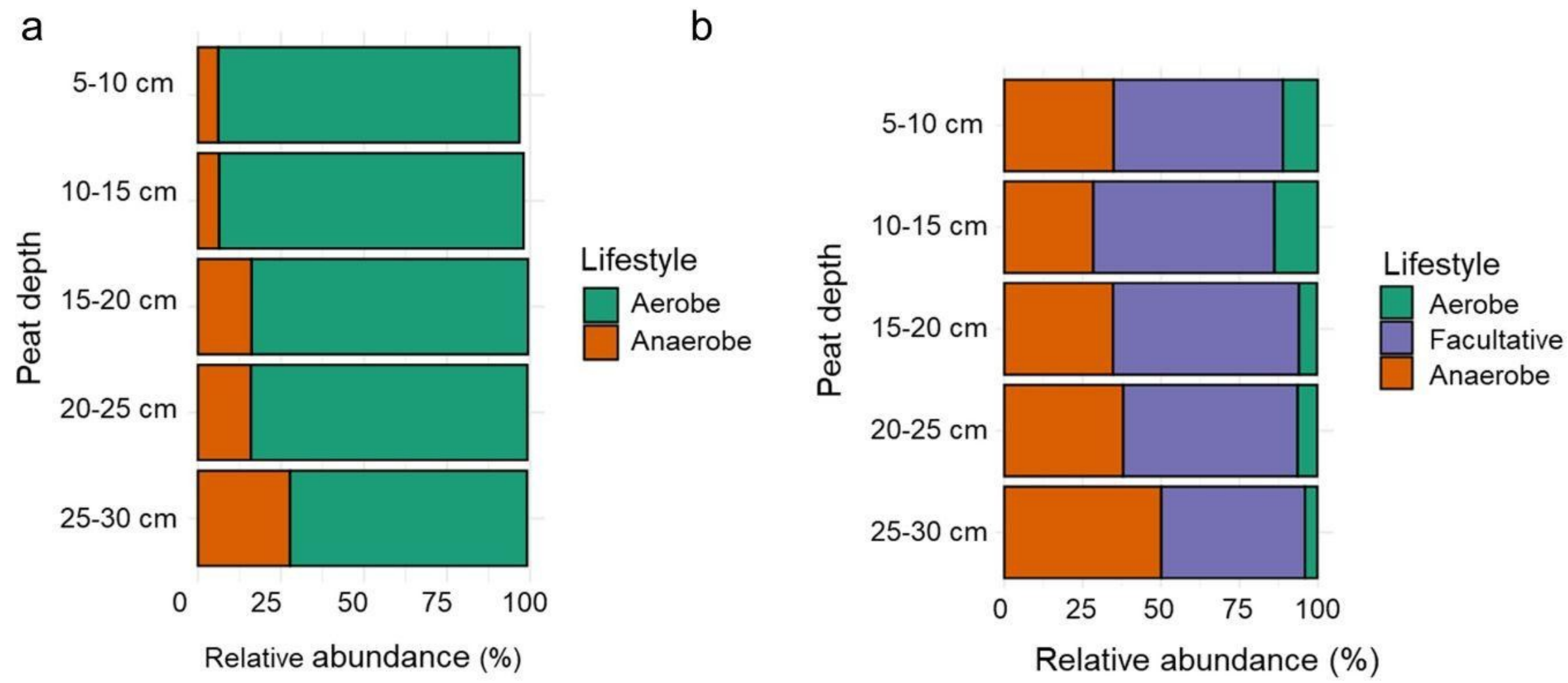

Supplementary Figure 3 | Respiratory potential of peatland MAGs and depth-resolved abundance.

Relative abundance of MAGs predicted as aerobic and anaerobic by (a) Aerobot[2] and (b) Oxyphen[3]. Abundances are read recruitment–based (CoverM) and expressed as fraction of total MAG abundance per sample and depth interval. Source data are provided as a Source Data file.

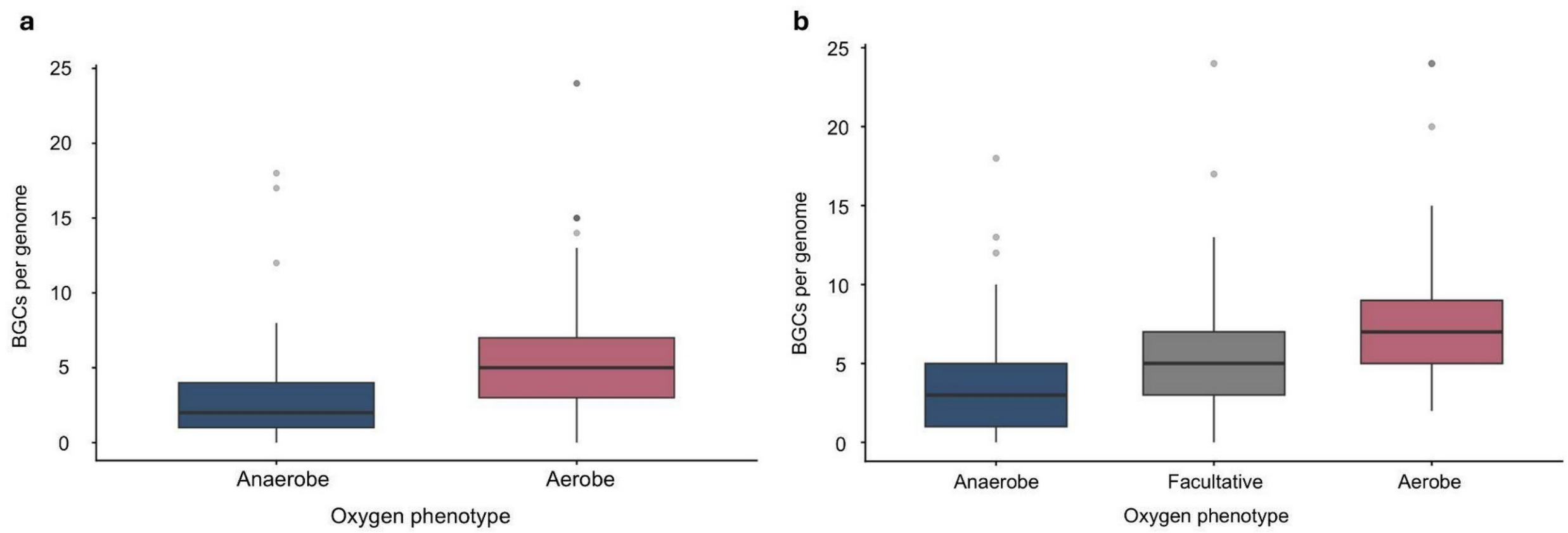

Supplementary Figure 4 | Genomic biosynthetic potential varies with predicted respiratory lifestyle. Number of biosynthetic gene clusters (BGCs) per metagenome-assembled genome (MAG) grouped by oxygen-utilization phenotype predicted using (a) Aerobot[2] and (b) Oxyphen[3]. Boxes indicate the median and interquartile range (IQR), whiskers extend to the most extreme values within 1.5 × IQR, and points beyond the whiskers indicate outliers. For Aerobot[2], n = 444 aerobic MAGs and n = 151 anaerobic MAGs. For Oxyphen[3], n = 58 aerobic MAGs, n = 265 facultative MAGs and n = 272 anaerobic MAGs. Aerobic and anaerobic MAGs were compared using two-sided Welch's t-tests. Using Aerobot[2], aerobic MAGs encoded a mean of 5 BGCs per MAG compared with 2.87 in anaerobic MAGs (mean difference = 2.13 BGCs per MAG, 95% CI = 1.58–2.68; $t = 7.62$, df = 290.17, $P = 3.565 \times 10^{-13}$). Using Oxyphen[3], aerobic MAGs encoded a mean of 8.03 BGCs per MAG compared with 2.75 in anaerobic MAGs (mean difference = 5.29 BGCs per MAG, 95% CI = 4.11–6.47; $t = 8.97$, df = 64.35, $P = 6.192 \times 10^{-13}$). Source data are provided as a Source Data file.

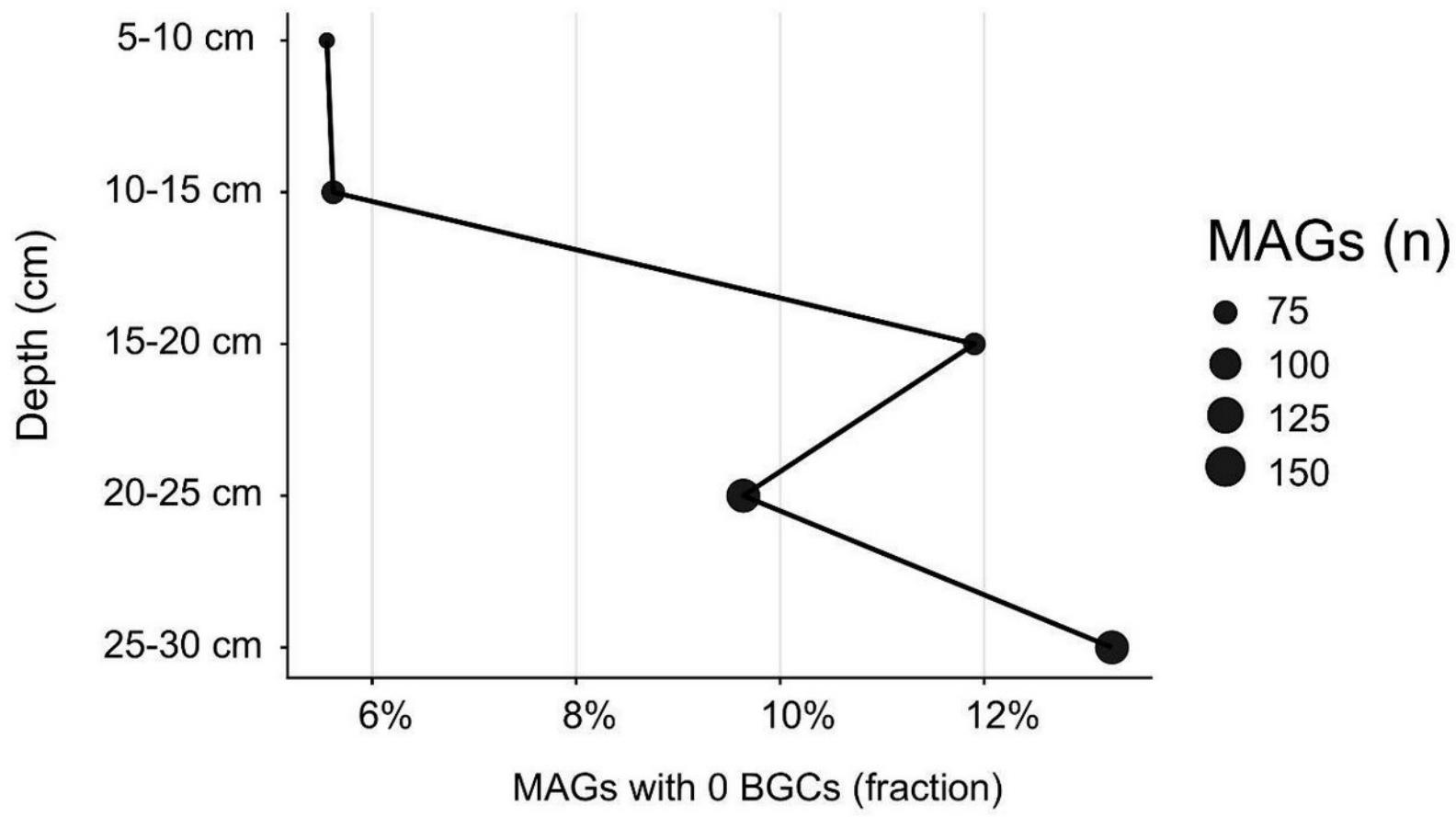

Supplementary Figure 5 | Frequency of MAGs lacking detectable BGCs. Proportion of metagenome-assembled genomes (MAGs) with no antiSMASH-detected biosynthetic gene clusters (BGCs) at each peat depth. Points represent the fraction of MAGs with zero detected BGCs, and point size indicates the total number of MAGs included at each depth. Sample sizes were n = 72, 89, 84, 166 and 166 MAGs for 5–10, 10–15, 15–20, 20–25 and 25–30 cm, respectively. The 5–10, 10–15 and 15–20 cm depth intervals each represent a single Core B metagenome, whereas the 20–25 and 25–30 cm values were calculated by pooling MAGs recovered from Cores B and C. Source data are provided as a Source Data file.

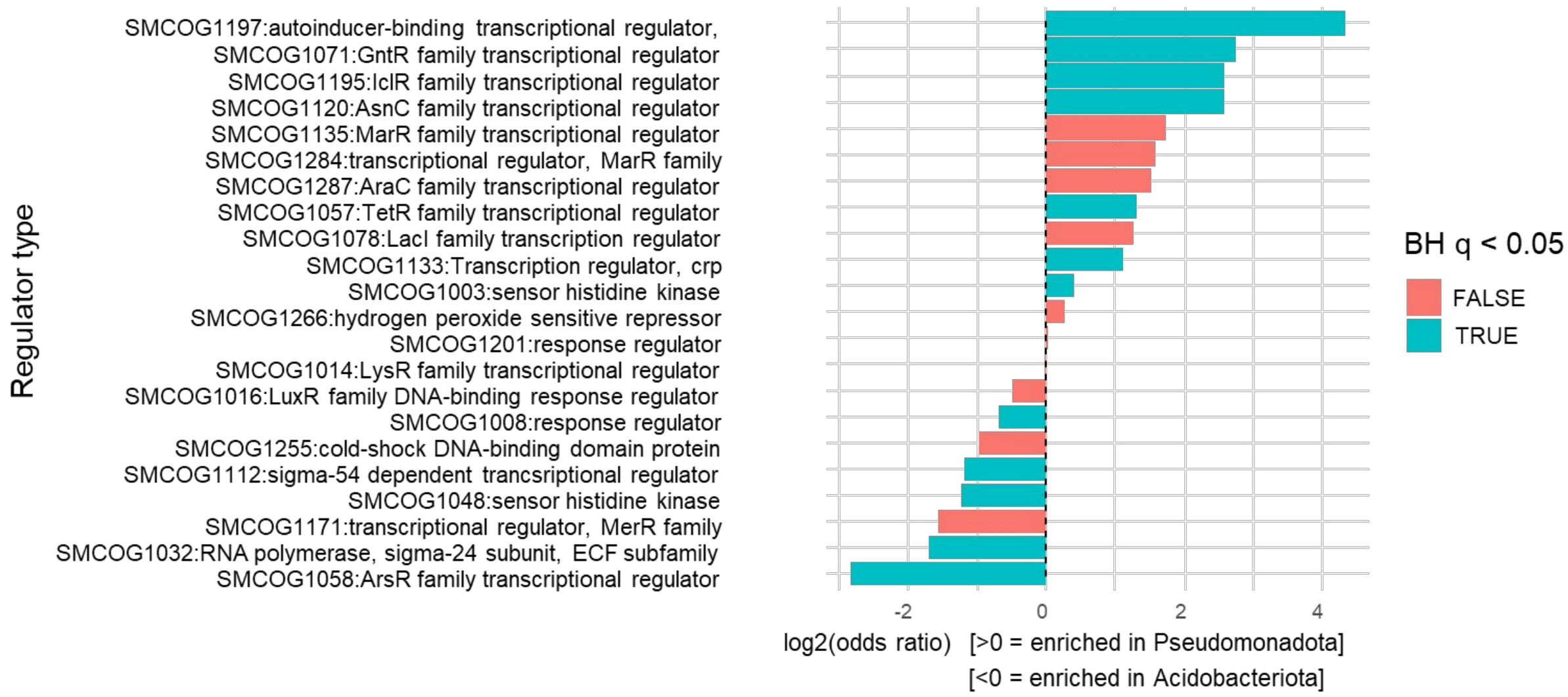

Supplementary Figure 6 | Differential enrichment of BGC-associated regulators in Pseudomonadota and Acidobacteriota. Enrichment of antiSMASH v7 SMCOG-annotated regulatory proteins in biosynthetic gene clusters (BGCs) from Pseudomonadota and Acidobacteriota MAGs. For each regulator type, a two-sided Fisher's exact test compared its frequency with that of all other regulator annotations within each phylum. The analysis comprised n = 912 SMCOG regulator annotations in Pseudomonadota BGCs and n = 1,189 regulator annotations in Acidobacteriota BGCs. Bars show the $\log_2$ odds ratio; positive values indicate enrichment in Pseudomonadota and negative values indicate enrichment in Acidobacteriota. P values were corrected across regulator types using the Benjamini–Hochberg procedure. Colours indicate whether the BH-adjusted P value (q) was < 0.05. Exact counts, odds ratios, raw P values and BH-adjusted P values are provided in Supplementary Table 6. Source data are provided as a Source Data file.

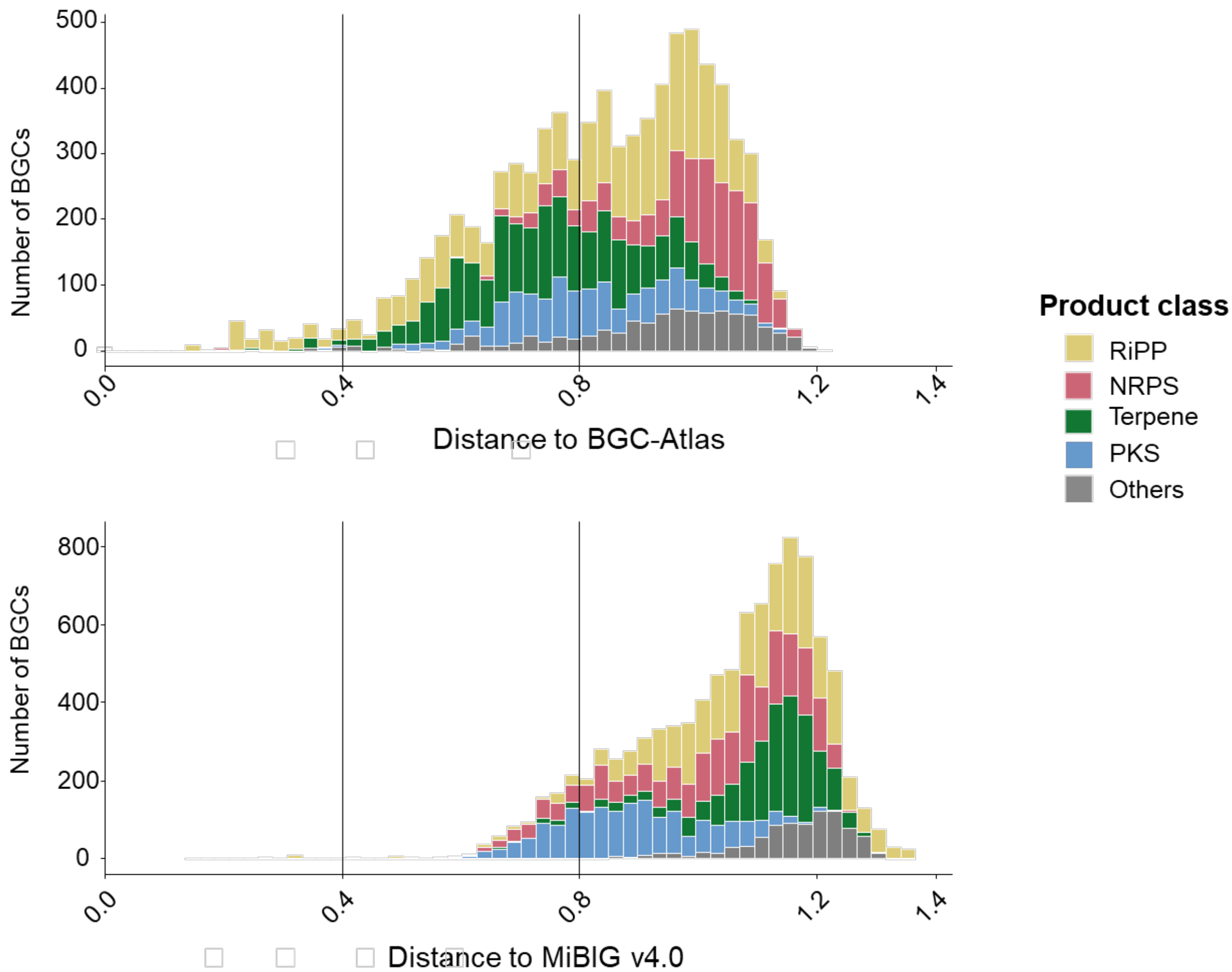

Supplementary Figure 7 | Distance of peatland BGCs to reference biosynthetic gene clusters. Distribution of BiG-SLiCE v2 distances for n = 8,150 biosynthetic gene clusters (BGCs) compared with (a) gene cluster families in BGC-Atlas[4] and (b) experimentally validated BGCs in MiBIG v4.0[5]. BiG-SLiCE distances quantify dissimilarity in biosynthetic feature space, with lower values indicating greater similarity to the reference BGC or gene cluster family. BGCs were queried against BGC-Atlas using the BiG-SLiCE 2.0[6] query function; 233 of 8,150 BGCs (2.9%) were assigned to an existing BGC-Atlas gene cluster family, whereas 7,917 (97.1%) were not. MiBIG v4.0 BGCs were clustered using BiG-SLiCE v2 with a distance threshold of 0.4 before querying the peatland BGCs. The vertical line at distance = 0.4 indicates the threshold used to define

a close reference match; 10 of 8,150 peatland BGCs (0.1%) had distances ≤0.4 to MiBIG BGCs, whereas 8,140 had distances >0.4. Histograms are coloured by BGC product class. Source data are provided as a Source Data file.

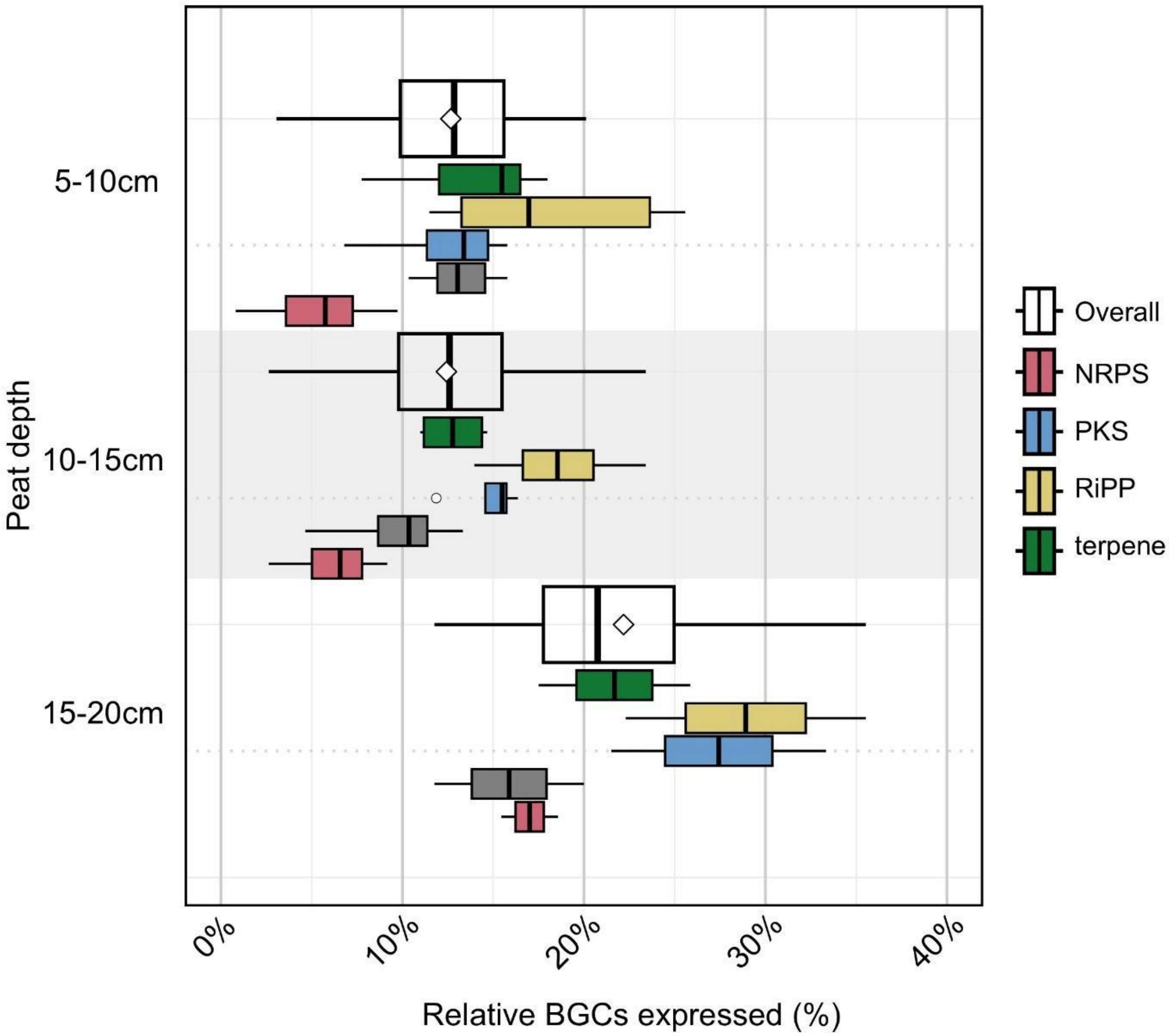

Supplementary Figure 8 | Depth-resolved expression of biosynthetic potential. Percentage of biosynthetic gene clusters (BGCs) expressed in active metagenome-assembled genomes (MAGs) within each metatranscriptome sample, shown overall and for major BGC product classes. Overall values combine all included BGC product classes within each sample. Boxplots summarize sample-level percentages at each peat depth, with n = 6, 4 and 2 independent metatranscriptome samples for 5–10, 10–15 and 15–20 cm, respectively. Boxes indicate the median and interquartile range (IQR), and whiskers

extend to the most extreme values within 1.5 × IQR. White diamonds indicate the mean. Colours denote BGC product class. Source data are provided as a Source Data file.

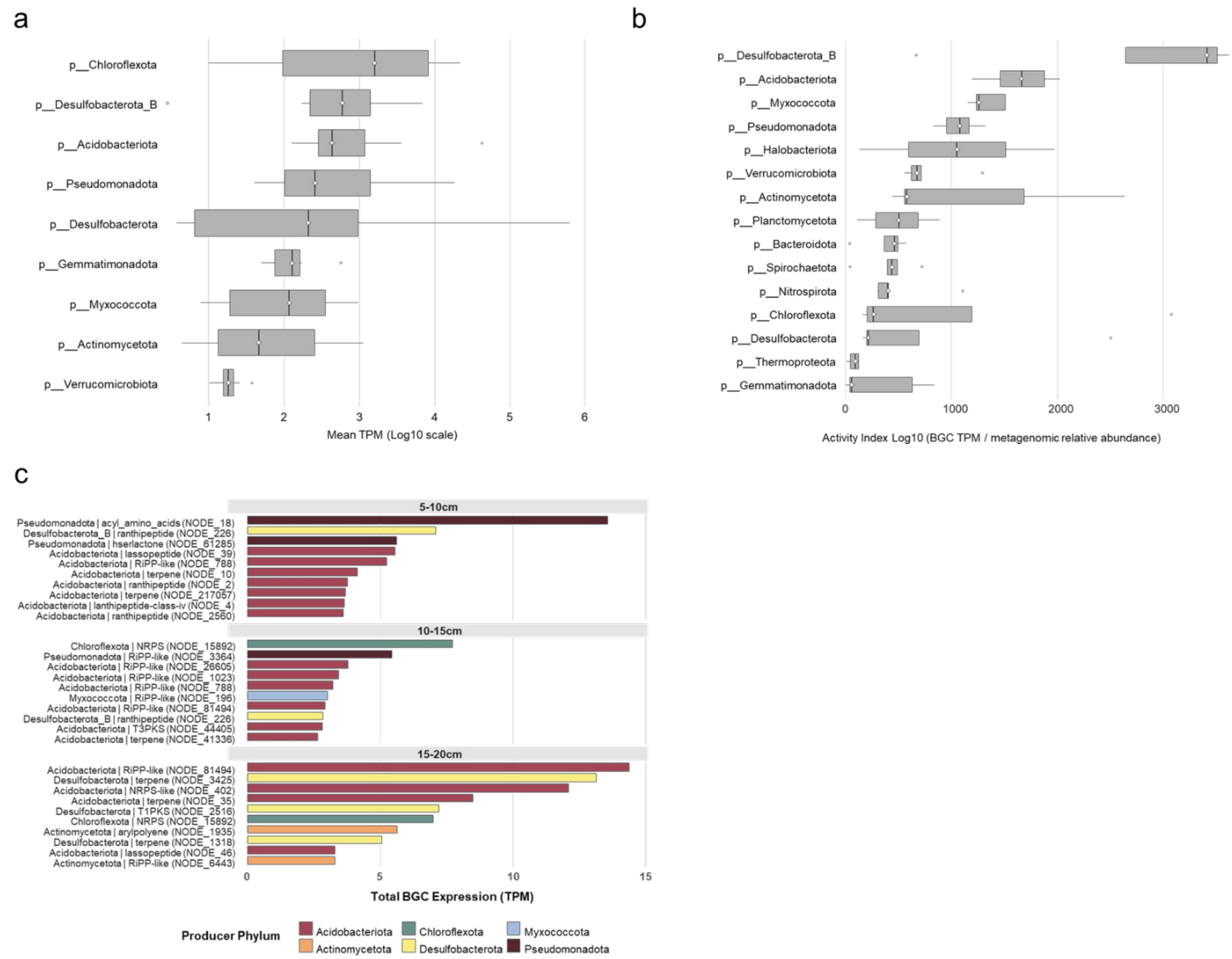

Supplementary Figure 9 | Phylum-resolved BGC transcriptional activity, abundance-corrected expression, and the most highly expressed clusters across peatland depths. (A) BGC transcriptional intensity by phylum. For each phylum in each metatranscriptome sample, transcriptional intensity was calculated as the mean transcripts per million (TPM) across actively expressed biosynthetic gene clusters (BGCs). Sample sizes were n = 12 metatranscriptome samples. Phylum–sample combinations lacking actively expressed BGCs were not included. (B) Abundance-normalized BGC transcriptional activity by phylum. For each phylum × sample observation, the activity index was calculated as total phylum-level BGC transcript

abundance (summed TPM of expressed BGCs) divided by the corresponding depth-matched metagenomic relative abundance. Sample sizes were n = 12 observations per phylum for Acidobacteriota, Actinomycetota, Bacteroidota, Chloroflexota, Desulfobacterota, Desulfobacterota_B, Gemmatimonadota, Myxococcota, Nitrospirota, Planctomycetota, Pseudomonadota, Spirochaetota, Thermoproteota and Verrucomicrobiota. (C) Most highly expressed individual BGCs at each peat depth. BGCs were ranked by their mean transcript abundance across metatranscriptome samples within each depth, and the ten BGCs with the highest mean expression are shown for each depth interval. Bars indicate mean BGC expression in transcripts per million (TPM), with individual points showing the underlying sample-level TPM values. Sample sizes were n = 6, 4 and 2 independent metatranscriptome samples for 5–10, 10–15 and 15–20 cm, respectively. Bars are coloured according to the phylum encoding each BGC, and labels indicate the predicted BGC product class and contig identifier. Source data are provided as a Source Data file.

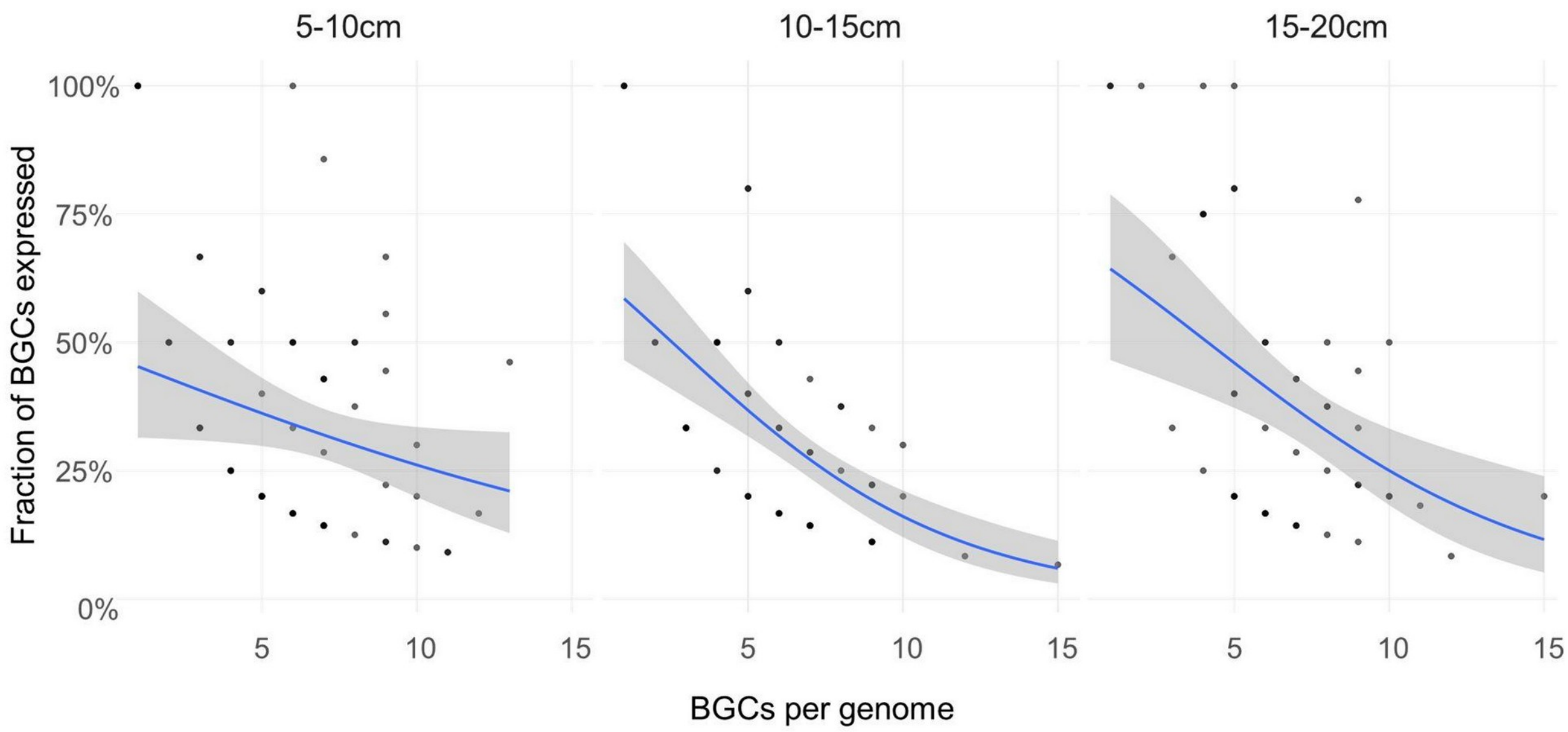

Supplementary Figure 10 | The fraction of BGCs expressed decreases with BGC repertoire size across active MAGs. Relationship between the number of biosynthetic gene clusters (BGCs) encoded per metagenome-assembled genome (MAG) and the fraction of those BGCs expressed at each peat depth, in MAGs expressing at least 1 BGC. Each point represents one active MAG × depth observation. Sample sizes were n = 73, 55 and 47 MAGs at 5–10, 10–15 and 15–20 cm, respectively, corresponding to 175 MAG × depth observations from 112 unique MAGs. BGC repertoire size was negatively associated with the fraction of BGCs expressed at all depths (two-sided Spearman's rank correlation: 5–10 cm, $\rho = -0.432$, $P = 1.37 \times 10^{-4}$; 10–15 cm, $\rho = -0.678$, $P = 1.33 \times 10^{-8}$; 15–20 cm, $\rho = -0.504$, $P = 3.09 \times 10^{-4}$). Source data are provided as a Source Data file.

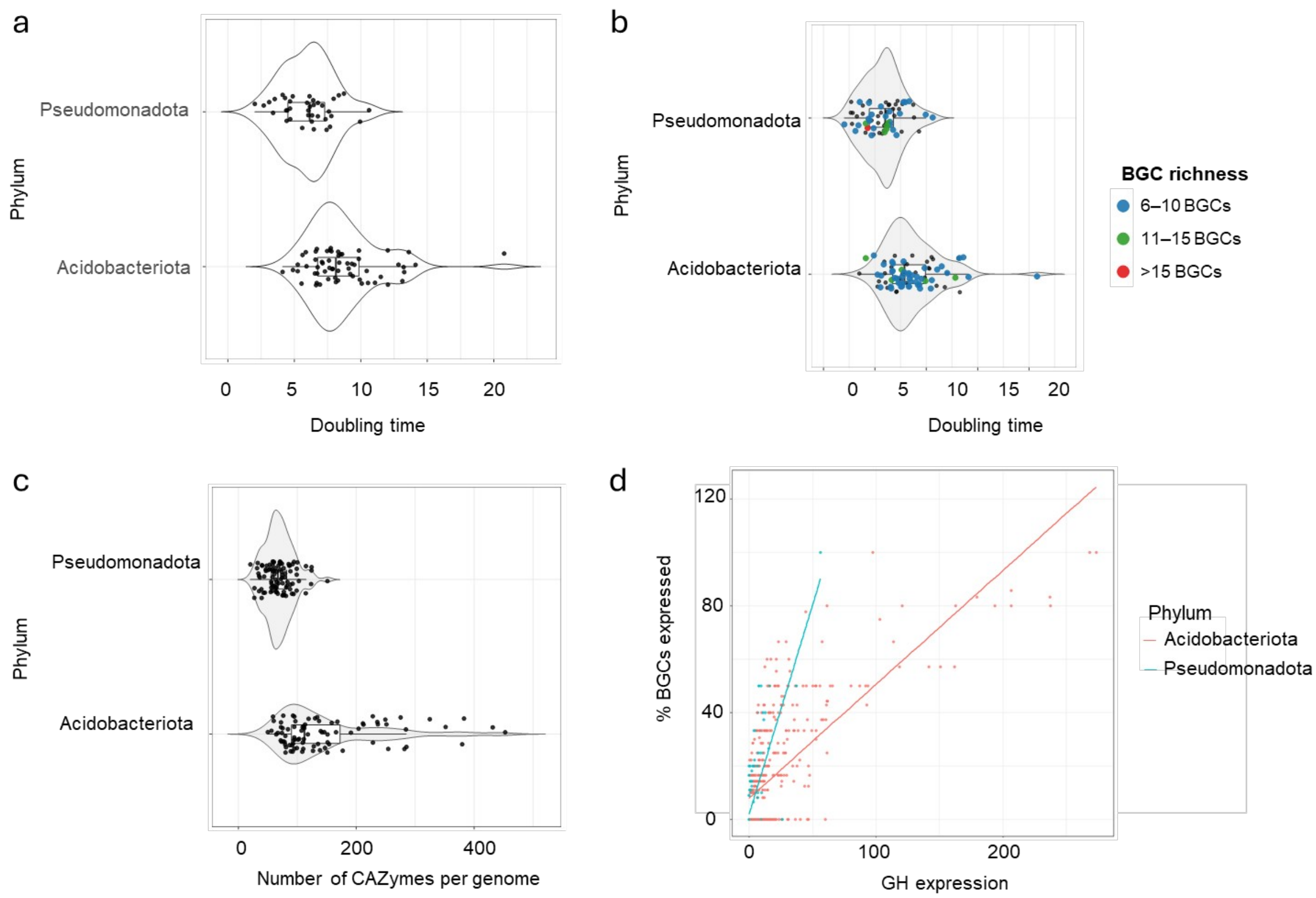

Supplementary Figure 11 | Predicted doubling times, complex-carbon degradation potential and BGC activity in Acidobacteriota and Pseudomonadota. (a) Predicted doubling times were higher in Acidobacteriota than in Pseudomonadota. Points represent individual MAGs (n = 81 Acidobacteriota and n = 64 Pseudomonadota MAGs). Mean predicted doubling times were 8.54 and 5.77, respectively (mean difference = 2.77, 95% CI 2.03–3.52; two-sided Welch's t-test, t = 7.35, df = 140.49, $P = 1.45 \times 10^{-11}$). (b) The same pattern was observed among BGC-rich MAGs, defined as encoding ≥5 BGCs per genome (n = 61 Acidobacteriota and n = 38 Pseudomonadota MAGs). Mean predicted doubling times were 8.72 and 5.98, respectively (mean difference = 2.74, 95% CI 1.79–3.68; two-sided Welch's t-test, t = 5.73, df = 96.06, $P = 1.17 \times 10^{-7}$). (c) Acidobacteriota encoded more carbohydrate-active enzymes associated with complex-carbon

degradation than Pseudomonadota. Values represent the number of GH-, AA- and PL-class CAZyme annotations per genome for the same MAGs (n = 81 Acidobacteriota and n = 64 Pseudomonadota MAGs). Mean counts were 152.6 and 69.9 per genome, respectively (mean difference = 82.7, 95% CI 61.8–103.7; two-sided Welch's t-test, $t = 7.84$, $df = 96.01$, $P = 6.22 \times 10^{-12}$). For a - c, violin plots show the distribution of MAG-level values; boxes indicate the median and interquartile range (IQR), whiskers extend to the most extreme values within 1.5 × IQR, and points represent individual MAGs. (d) Glycoside hydrolase (GH) expression was positively associated with BGC deployment across Acidobacteriota and Pseudomonadota. Each point represents one MAG × metatranscriptome observation; the analysis comprised n = 645 observations from 102 unique MAGs across 12 metatranscriptome samples (Acidobacteriota: 436 observations from 64 MAGs; Pseudomonadota: 209 observations from 38 MAGs). Source data are provided as a Source Data file.

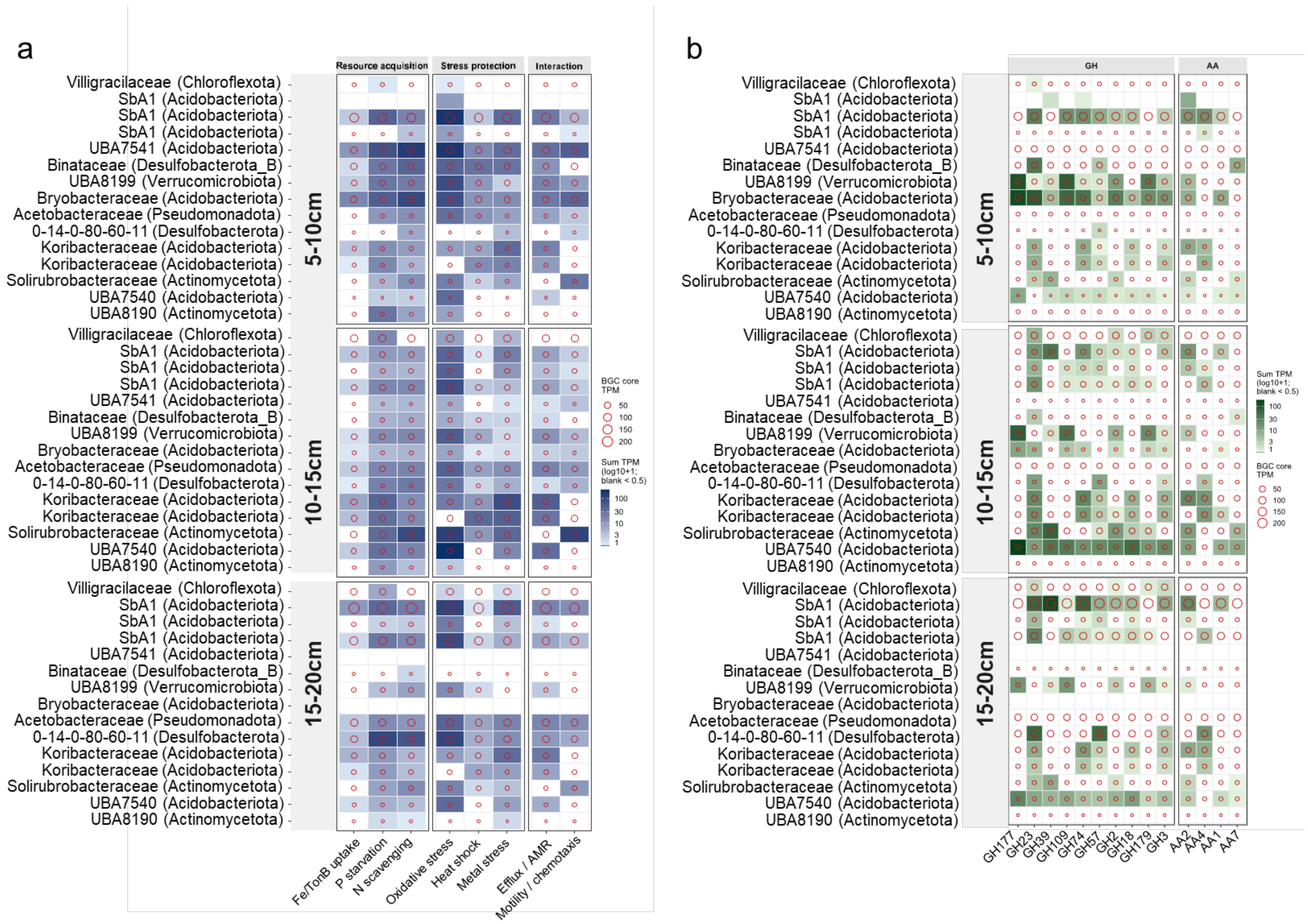

Supplementary Figure 12 | Trait module and CAZyme expression across focal MAGs and depths.(A) Expression of curated KEGG-derived functional trait modules across 15 focal MAGs at three peat depths. Modules are grouped into three categories: resource acquisition (Fe/TonB uptake, phosphorus starvation response, nitrogen scavenging), stress protection (oxidative stress, heat shock, metal stress), and interaction/defence (efflux/AMR, motility/chemotaxis). Heatmap fill indicates summed module TPM ($\log_{10}$(TPM + 1) and cells with summed TPM < 0.5 are shown as blank. Overlaid open circles indicate BGC core-gene TPM per MAG × depth observation, with circle area proportional to expression intensity (scale shown). (B) Expression of glycoside hydrolase (GH) and auxiliary activity (AA) CAZyme families across the same 15 focal

MAGs and depths. Heatmap fill indicates summed family TPM (log_{10}(TPM + 1); blank < 0.5). Overlaid circles indicate BGC core-gene TPM as in (A). Source data are provided as a Source Data file.

Supplementary Tables

Supplementary Table 1. Sequencing depth, assembly size, number of MAGs and output of Nonpareil3 on seven metagenome samples

						Nonpareil3 stats		
Sample	Peat Core	Depth	Raw reads	Assembly size (MB)	MAGs	kappa	C	diversity
no001_B5-10	B	5-10 cm	601,849,164	1372	73	0.73238	0.752234	22.6235
no002_B10-15	B	10-15 cm	541,368,064	1436	92	0.74132	0.760623	22.4301
no003_B15-20	B	15-20 cm	480,127,122	978	89	0.88354	0.892985	20.4096
no004_B20-25	B	20-25 cm	511,682,362	1104	72	0.85101	0.862883	21.0760
no006_C20-25	C	20-25 cm	493,097,598	963	65	0.84136	0.853934	20.9481
no005_B25-30	B	25-30 cm	488,981,894	1002	88	0.88064	0.890306	20.4338
no007_C25-30	C	25-30 cm	529,909,558	1131	94	0.88278	0.892283	20.3250

Supplementary Table 2. Relative abundance of bacterial and archaeal phyla across peatland metagenomes, from depths of 5 – 30 cm as determined through the abundance of single copy gene markers extracted from metagenome reads.

	Core 1					Core 2	
**taxonomy**	5-10cm	10-15 cm	15-20cm	20-25cm	25-30cm	20-25cm	25-30cm
p__Acidobacteriota	41.14	38.33	42.96	39.65	32.64	36.27	28.96
p__Pseudomonadota	15.71	17.74	17.33	18.03	18.19	21.49	20.14
Archaea; p__Thermoproteota	1.76	2.94	7.41	7.78	7.83	9.29	9.9
p__Desulfobacterota	0.95	1.94	5.73	5.15	11.16	7.66	13.44
p__Actinomycetota	10.05	9.33	3.11	4.35	4.68	2.72	3.33
p__Verrucomicrobiota	6.44	6.06	2.7	3.28	3.1	2.24	2.38
p__Patescibacteria	4.73	4.39	2.05	2.69	2.46	1.71	1.42
p__Chloroflexota	1.88	2.98	2.47	2.73	3.27	2.49	3.13
p__Planctomycetota	2.75	3.11	1.86	2.06	1.67	1.7	1.52
p__Desulfobacterota_B	1.2	1.25	3.16	3.23	1.32	3.16	1.19
p__Nitrospirota	0.36	0.41	1.38	1.25	3.1	1.68	3.73
p__Bacteroidota	1.92	1.62	1.06	1.09	1.14	0.9	1.18
p__Myxococcota	2.98	1.96	0.6	0.93	1	0.58	0.55
p__Spirochaetota	0.84	0.7	0.82	0.71	1	0.8	1.26
p__Desulfobacterota_F	0.05	0.11	0.88	0.78	1.21	1.17	1.45
p__Chlamydiota	1.04	1.08	0.67	0.69	0.55	0.66	0.8
p__Gemmatimonadota	0.28	0.32	1.18	0.94	0.8	0.91	0.6
Archaea; p__Halobacteriota	0.08	0.16	0.74	0.56	0.92	0.88	1.4
unassigned	0.31	0.54	0.41	0.63	0.43	0.6	0.49
Others	5.51	5.06	3.47	3.44	3.39	3.03	2.99

Supplementary Table 3. Relative abundance of MAGs grouped to phylum level, for metagenomes across peat depths from 5 – 30 cm, determined by alignment of metagenome reads to MAG sequences using CoverM.

	Core 1					Core 2	
**taxonomy**	5-10cm	10-15 cm	15-20cm	20-25cm	25-30cm	20-25cm	25-30cm
p__Acidobacteriota	50.70	47.56	45.87	42.93	30.94	36.13	26.20
p__Pseudomonadota	14.90	17.75	19.07	20.08	20.55	23.65	22.47
p__Desulfobacterota	0.71	1.41	8.91	8.78	19.02	12.30	20.67
p__Thermoproteota	3.39	4.24	9.04	9.64	10.30	11.19	12.48
p__Actinomycetota	8.56	9.48	2.16	2.92	3.96	2.12	3.34
p__Verrucomicrobiota	8.25	7.41	1.38	2.14	1.84	1.19	1.11
p__Chloroflexota	1.99	2.23	1.86	2.22	2.68	2.19	2.64
p__Desulfobacterota_B	1.88	1.51	2.50	2.55	0.91	2.35	0.82
p__Nitrospirota	0.20	0.58	1.61	1.52	2.11	1.94	2.35
p__Gemmatimonadota	0.33	0.83	2.06	1.70	1.46	1.43	0.95
p__Halobacteriota	0.09	0.10	1.20	1.06	1.54	1.46	2.11
p__Spirochaetota	2.48	1.44	0.53	0.53	0.84	0.51	0.82
p__Myxococcota	2.38	1.64	0.61	0.78	0.65	0.51	0.25
p__Bacteroidota	1.60	0.77	0.76	0.68	0.88	0.69	0.91
p__Planctomycetota	0.56	0.77	1.08	1.04	0.65	0.78	0.63
Other	1.97	2.27	1.36	1.44	1.68	1.57	2.25

Supplementary Table 4. The total number of BGCs detected by antiSMASH v7 in metagenome assemblies.

Core	Peat Depth	Assembly size (MB)	Total BGCs
B	5-10 cm	1372	1918
B	10-15 cm	1436	1960
B	15-20 cm	978	1110
B	20-25 cm	1104	1302
C	20-25 cm	963	1119
B	25-30 cm	1002	1107
C	25-30 cm	1131	1178

Supplementary Table 5. Biosynthetic gene clusters, deduplicated to Gene Cluster families (GCFs) using BiG-SCAPE v1, and assigned to broader BiG-SCAPE categories.

Phylum	RiPPs	NRPS	PKSother	Terpene	Other	PKSI	PKS-NRP Hybrid
Acidobacteriota	607	540	257	248	120	57	65
Pseudomonadota	268	197	80	143	187	33	15
Actinomycetota	95	62	15	37	108	8	6
Myxococcota	92	55	26	10	43	3	7
Verrucomicrobiota	31	54	21	66	41	7	8
Desulfobacterota	35	9	7	16	24	10	0
Desulfobacterota B	35	8	14	6	6	1	2
Planctomycetota	15	17	10	29	11	1	1
Nitrospirota	10	17	6	12	16	4	1
Thermoproteota	5	0	0	4	15	0	0
Bacteroidota	8	6	14	10	5	0	1
Chloroflexota	3	6	9	7	13	2	0
Bdellovibrionota	12	1	2	3	0	0	0
Spirochaetota	0	0	8	0	2	0	0
Desulfobacterota G	7	0	0	4	6	0	0
Gemmatimonadota	7	1	0	0	1	0	0
Halobacteriota	7	1	0	0	0	0	0
Patescibacteria	6	1	0	1	3	0	0
Thermoplasmatota	1	0	0	1	6	0	0
Unassigned	129	217	87	20	49	71	52
Other Phyla	23	12	10	15	6	1	0

Supplementary Table 6. Regulatory genes and transcription factors associated with biosynthetic gene clusters that were differentially enriched between Pseudomonadota and Acidobacteriota. Regulator annotations were obtained from antiSMASH v7 BGC outputs. Enrichment between phyla was assessed using two-sided Fisher's exact tests on regulator counts. Odds ratios (ORs) with 95% confidence intervals are reported as effect-size estimates. P values were adjusted for multiple comparisons across the tested regulator types using the Benjamini–Hochberg procedure; both raw and BH-adjusted P values are reported. An OR > 1 indicates enrichment in the focal phylum relative to the comparison phylum.

regulator_info	# in Pseudo.	# in Acido	odds_ratio	log2_OR	p_value	p_adj
SMCOG1015:AraC family transcriptional regulator	7	0	Inf	Inf	0.004076	0.010347
SMCOG1031:LysR family transcriptional regulator	42	0	Inf	Inf	3.05E-15	5.03E-14
SMCOG1125:LysR family transcriptional regulator	2	0	Inf	Inf	0.208155	0.264197
SMCOG1126:extracytoplasmic-function sigma-70 factor	4	0	Inf	Inf	0.043235	0.075092
SMCOG1136:GntR family transcriptional regulator	12	0	Inf	Inf	7.87E-05	0.000289
SMCOG1149:LuxR family transcriptional regulator	20	0	Inf	Inf	1.38E-07	1.14E-06
SMCOG1215:TetR family transcriptional regulator	1	0	Inf	Inf	0.456364	0.537857
SMCOG1260:AraC family transcriptional regulator	4	0	Inf	Inf	0.043235	0.075092
SMCOG1197:autoinducer-binding transcriptional regulator,	48	3	19.95	4.32	1.08E-13	1.19E-12
SMCOG1071:GntR family transcriptional regulator	22	4	6.67	2.74	7.46E-05	0.000289
SMCOG1120:AsnC family transcriptional regulator	10	2	6.00	2.59	0.015996	0.032991
SMCOG1195:IclR family transcriptional regulator	10	2	6.00	2.59	0.015996	0.032991
SMCOG1135:MarR family transcriptional regulator	11	4	3.30	1.72	0.037355	0.072513
SMCOG1284:transcriptional regulator, MarR family	5	2	2.99	1.58	0.257105	0.314239
SMCOG1287:AraC family transcriptional regulator	12	5	2.88	1.53	0.049318	0.081375
SMCOG1057:TetR family transcriptional	77	39	2.46	1.30	5.16E-06	2.84E-05

regulator						
SMCOG1078:LacI family transcription regulator	10	5	2.40	1.26	0.121438	0.182157
SMCOG1133:Transcription regulator, crp	44	25	2.15	1.10	0.002943	0.008094
SMCOG1003:sensor histidine kinase	272	262	1.32	0.41	0.005166	0.012178
SMCOG1266:hydrogen peroxide sensitive repressor	1	1	1.19	0.25	1	1
SMCOG1201:response regulator	6	7	1.02	0.03	1	1
SMCOG1014:LysR family transcriptional regulator	51	62	0.98	-0.03	0.923129	1
SMCOG1016:LuxR family DNA-binding response regulator	52	86	0.71	-0.50	0.063452	0.09971
SMCOG1008:response regulator	135	240	0.62	-0.69	4.01E-05	0.000189
SMCOG1255:cold-shock DNA-binding domain protein	6	14	0.51	-0.98	0.181357	0.249365
SMCOG1112:sigma-54 dependent trancsriptional regulator	58	145	0.44	-1.17	2.64E-07	1.74E-06
SMCOG1048:sensor histidine kinase	21	57	0.43	-1.23	0.000712	0.002137
SMCOG1171:transcriptional regulator, MerR family	2	7	0.34	-1.56	0.193757	0.255759
SMCOG1032:RNA polymerase, sigma-24 subunit, ECF	56	192	0.31	-1.69	2.45E-15	5.03E-14
SMCOG1058:ArsR family transcriptional regulator	3	25	0.14	-2.83	0.000154	0.000508

References

1. Rodriguez-R, L. M., Gunturu, S., Tiedje, J. M., Cole, J. R. & Konstantinidis, K. T. Nonpareil 3: Fast estimation of metagenomic coverage and sequence diversity. mSystems 3, (2018).

2. Flamholz, A. I. et al. Annotation-free prediction of microbial dioxygen utilization. mSystems 9, e0076324 (2024).

3. Jabłońska, J. & Tawfik, D. S. The number and type of oxygen-utilizing enzymes indicates aerobic vs. anaerobic phenotype. Free Radic. Biol. Med. 140, 84–92 (2019).

4. Bağcı, C. et al. BGC Atlas: a web resource for exploring the global chemical diversity encoded in bacterial genomes. Nucleic Acids Res. 53, D618–D624 (2025).

5. Zdouc, M. M. et al. MIBiG 4.0: advancing biosynthetic gene cluster curation through global collaboration. Nucleic Acids Res. 53, D678–D690 (2025).

6. Draisma, A. et al. BiG-SCAPE 2.0 and BiG-SLiCE 2.0: scalable, accurate and interactive sequence clustering of metabolic gene clusters. bioRxiv 2025.08.20.671210 (2025) doi:10.1101/2025.08.20.671210.
