# Supplementary material for: High diversity but limited expression of biosynthetic gene clusters in a peatland microbial community
Source: Nat Commun. 2026 Oct 6;17:10529. doi: 10.1038/s41467-026-78121-8 (PMC13642348; doi:10.1038/s41467-026-78121-8)
Supplement: Supplementary file 2 [file 41467_2026_78121_MOESM2_ESM.pdf]

Description Of Additional Supplementary Files

Supplementary data 1: List of MAGs used, with CheckM2 stats and GTDB taxonomy.

Supplementary data 2: List of antiSMASH detected biosynthetic gene clusters, including predicted product, assigned BiG-SCAPE class, sample of origin and taxonomy.

Supplementary data 3: Per-BGC summary table used to assess potential overestimation caused by short-read assembly fragmentation. For each antiSMASH-predicted BGC, the table reports contig-edge status, predicted product, broader product class, number of biosynthetic genes, the minimum and maximum distance of biosynthetic core genes to the nearest contig end, and whether the BGC contains an anchored biosynthetic core gene located entirely more than 5 kb or 10 kb from a contig end.

Supplementary data 4: All MAGs recovered encoding at least 5 BGCs, indicated with the number of BGCs encoded, genome size and taxonomic affiliation.

Supplementary data 5: Gene annotations used to do the functional analyses related to oxygen, energy, carbohydrate-active enzyme and resource and stress related metabolism in 15 focal MAGs with active BGC expression.
