# Supplementary material for: High diversity but limited expression of biosynthetic gene clusters in a peatland microbial community
Source: Nat Commun. 2026 Oct 6;17:10529. doi: 10.1038/s41467-026-78121-8 (PMC13642348; doi:10.1038/s41467-026-78121-8)
Supplement: Supplementary file 3 [file 41467_2026_78121_MOESM8_ESM.pdf]

nature portfolio

Corresponding author(s): Carl-Eric Wegner | carl-eric.wegner@hhu.de

Last updated by author(s): 24 August 2026

Reporting Summary

Nature Portfolio wishes to improve the reproducibility of the work that we publish. This form provides structure for consistency and transparency in reporting. For further information on Nature Portfolio policies, see our Editorial Policies and the Editorial Policy Checklist.

Statistics

For all statistical analyses, confirm that the following items are present in the figure legend, table legend, main text, or Methods section.

n/a	Confirmed	
☐	☒	The exact sample size (*n*) for each experimental group/condition, given as a discrete number and unit of measurement
☒	☐	A statement on whether measurements were taken from distinct samples or whether the same sample was measured repeatedly
☐	☒	The statistical test(s) used AND whether they are one- or two-sided<br>*Only common tests should be described solely by name; describe more complex techniques in the Methods section.*
☒	☐	A description of all covariates tested
☐	☒	A description of any assumptions or corrections, such as tests of normality and adjustment for multiple comparisons
☐	☒	A full description of the statistical parameters including central tendency (e.g. means) or other basic estimates (e.g. regression coefficient) AND variation (e.g. standard deviation) or associated estimates of uncertainty (e.g. confidence intervals)
☐	☒	For null hypothesis testing, the test statistic (e.g. *F*, *t*, *r*) with confidence intervals, effect sizes, degrees of freedom and *P* value noted<br>*Give P values as exact values whenever suitable.*
☒	☐	For Bayesian analysis, information on the choice of priors and Markov chain Monte Carlo settings
☒	☐	For hierarchical and complex designs, identification of the appropriate level for tests and full reporting of outcomes
☐	☒	Estimates of effect sizes (e.g. Cohen's *d*, Pearson's *r*), indicating how they were calculated

Our web collection on statistics for biologists contains articles on many of the points above.

Software and code

Policy information about availability of computer code

Data collection

Provide a description of all commercial, open source and custom code used to collect the data in this study, specifying the version used OR state that no software was used.

Data analysis

Metagenome reads were trimmed using TrimGalore (v0.6.6) and reads were merged using Flash (v1.2.11). Metagenome sequences were assembled using SPAdes74 (v3.15.3). Assemblies >2000 bp were binned using MetaBat2 (v2.15), Concoct (v1.1.0), and Maxbin (v2.2.5). Bins were then evaluated with DAS Tool (v1.1.2) to select the best non-overlapping bins. Bin quality, percent of binned population, and estimated percent of community were assessed using CheckM (v1.2.2). Gene calling was done using Prodigal v2.6.3, as implemented in Anvi'o v8. Contig-level taxonomy was determined using MMseqs2, using GTDB Release 220 as reference. MAG taxonomic annotation was performed using GTDB-tk, using GTDB Release 220. Biosynthetic gene clusters (BGCs) were identified from metagenome contigs longer than 5 kb using AntiSMASH (v7.0.1). The BGCs identified within each sample were further clustered into biosynthetic gene cluster families (GCFs) as a means to dereplicate them using BiG-SCAPE (v1.1.9) BGC novelty was assessed by comparing GCFs against the BGC-Atlas4 and MiBIG 4.0 databases using BiG-SLiCE 2.0.
Raw metatranscriptome reads were quality filtered and sequencing adapters removed using fastp, followed by rRNA filtering using Ribodetector v0.3.1. ribosomal RNA-depleted QC-reads were aligned to whole assemblies using bowtie2 (v2.4.5), SAM files were converted to BAM with samtools (v1.7). Further analysis were performed in R v4.3.0 using packages dplyr (v1.1.2), forcats (v1.0.0) and stringr (v1.5.0) were used for processing large data tables, and ggplot2 (v3.5.2). More detailed information is available in the Zenodo repository for this paper under the doi 10.5281/zenodo.17812908.

All software used in the analysis are: Trim Galore v0.6.6, FLASH v1.2.11, SPAdes v3.15.3, Nonpareil v3.5.5, MetaBAT2 v2.15, CONCOCT v1.1.0, MaxBin v2.2.5, DAS Tool v1.1.2, CheckM2 v1.0.2, Prodigal v2.6.3, anvi'o v8, METABOLIC v4.0, antiSMASH v7.0.1, BiG-SCAPE v1.1.9, BiG-SLiCE v2.0, Bowtie2 v2.4.5, fastp v0.23.4, RiboDetector v0.3.1, dRep v2.6.2, SAMtools v1.7, BBTools v39.01, CoverM v0.7.0, MMseqs2 v13.45111,

GTDB-Tk v2.5.2, featureCounts v2.0.6, SingleM v0.21.4, dbCAN v5.2.1, R v4.3.0, dplyr v1.1.2, forcats v1.0.0, stringr v1.5.0, ggplot2 v3.5.2, tidyverse v2.0.0, lme4 v1.1.34, lmerTest v3.1.3, iTOL v6, GTDB Release 220, MiBIG v4.0,

For manuscripts utilizing custom algorithms or software that are central to the research but not yet described in published literature, software must be made available to editors and reviewers. We strongly encourage code deposition in a community repository (e.g. GitHub). See the Nature Portfolio guidelines for submitting code & software for further information.

Data

Policy information about availability of data

All manuscripts must include a data availability statement. This statement should provide the following information, where applicable:

- Accession codes, unique identifiers, or web links for publicly available datasets
- A description of any restrictions on data availability
- For clinical datasets or third party data, please ensure that the statement adheres to our policy

Raw sequencing data generated in this study is available in the NCBI Sequence Read Archive under BioProject PRJNA1374247 (https://www.ncbi.nlm.nih.gov/bioproject/PRJNA1374247). Metagenome assembled genomes (MAGs) and Biosynthetic gene clusters generated in this study have been deposited in Zenodo under accession https://doi.org/10.5281/zenodo.17812908. Source data are provided with this paper. Code used for data analysis and to generate figures, along with additional data related to this manuscript are available at Zenodo (https://doi.org/10.5281/zenodo.17812908).

Research involving human participants, their data, or biological material

Policy information about studies with human participants or human data. See also policy information about sex, gender (identity/presentation), and sexual orientation and race, ethnicity and racism.

Reporting on sex and gender	N/A
Reporting on race, ethnicity, or other socially relevant groupings	N/A
Population characteristics	N/A
Recruitment	N/A
Ethics oversight	N/A

Note that full information on the approval of the study protocol must also be provided in the manuscript.

Field-specific reporting

Please select the one below that is the best fit for your research. If you are not sure, read the appropriate sections before making your selection.

☐ Life sciences ☐ Behavioural & social sciences ☒ Ecological, evolutionary & environmental sciences

For a reference copy of the document with all sections, see nature.com/documents/nr-reporting-summary-flat.pdf

Ecological, evolutionary & environmental sciences study design

All studies must disclose on these points even when the disclosure is negative.

Study description	We analyzed data from two parallel peat cores (B, and C) collected in July 2020 using a soil corer. Cores were sectioned in the field at 5 cm intervals down to 30 cm depth. This yielded a total of seven metagenomes. The study aim was to sequence metatranscriptomes from two peat cores, with three independent RNA extractions and metatranscriptomes from each depth. Despite multiple attempts, RNA could not be recovered from the deeper 20-30 cm layers, as well as some replicates of the 10-15 cm and 15 - 20 cm layers likely due to inhibitory compounds and high iron content. We thus have 12 metatranscriptomes from depths of 5 -20 cm, six replicates of the 5 - 10 cm, four replicates of the 10 - 15 cm and two replicates at 15 - 20 cm.
Research sample	Peat soil cores
Sampling strategy	Two parallel peat cores collected in July 2020 using a soil corer.
Data collection	Except for field samples, no additional data were collected from the study sites.
Timing and spatial scale	A single sampling expedition was undertaken on 29 July 2020.
Data exclusions	No data were excluded from the analyses performed.

Reproducibility	A single experiment was performed.
Randomization	N/A
Blinding	N/A

Did the study involve field work? ☒ Yes ☐ No

Field work, collection and transport

Field conditions	The conditions on the day of sampling were not recorded.
Location	Peat soil cores were collected from the Schlöppnerbrunnen fen, located in the northern Fichtelgebirge, Northern Bavaria, Germany (50°07′55″N, 11°52′52″E).
Access & import/export	No sampling, import and export permits were required to sample from the study area.
Disturbance	Two peat soil cores were collected, which cause minimal disturbance.

Reporting for specific materials, systems and methods

We require information from authors about some types of materials, experimental systems and methods used in many studies. Here, indicate whether each material, system or method listed is relevant to your study. If you are not sure if a list item applies to your research, read the appropriate section before selecting a response.

Materials & experimental systems

n/a	Involved in the study	
☒	☐	Antibodies
☒	☐	Eukaryotic cell lines
☒	☐	Palaeontology and archaeology
☒	☐	Animals and other organisms
☒	☐	Clinical data
☒	☐	Dual use research of concern
☒	☐	Plants

Methods

n/a	Involved in the study	
☒	☐	ChIP-seq
☒	☐	Flow cytometry
☒	☐	MRI-based neuroimaging

Plants

Seed stocks	NA
Novel plant genotypes	NA
Authentication	NA
