# Supplementary material for: High diversity but limited expression of biosynthetic gene clusters in a peatland microbial community
Source: Nat Commun. 2026 Oct 6;17:10529. doi: 10.1038/s41467-026-78121-8 (PMC13642348; doi:10.1038/s41467-026-78121-8)
Supplement: Supplementary file 4 [file 41467_2026_78121_MOESM9_ESM.pdf]

nature portfolio

Peer Review File

High diversity but limited expression of biosynthetic gene clusters in a peatland microbial community

Corresponding Author: Dr Carl-Eric Wegner

This file contains all reviewer reports in order by version, followed by all author rebuttals in order by version.

Version 0:

Reviewer comments:

Reviewer #1

(Remarks to the Author)
This original metagenomic study has led to the identification of a significant number of new secondary metabolite synthesis gene clusters (BGCs) at different depths in peatland cores.
The strengths and originality of this study are:
- The exploration of an ecosystem that has been understudied in functional metagenomics and which is characterised by strong environmental gradients over short distances, allowing hypotheses of functional adaptation of communities to be tested.
- The reconstruction of a large number of metagenome assembled genomes (MAGs) from metagenomic data, allowing BGCs, the overwhelming majority of which are new, to be associated with bacterial phyla.
- This association reveals the significant synthesis potential of understudied and often neglected bacterial phyla, such as acidobacteria.
- The association of metagenomic data with metatranscriptomic data reveals that a minority (and which ones) of BGCs are transcriptionally active in the samples studied.

This study confirms a well-known fact acquired in the laboratory, namely that within a microbial genome, only a very limited number of BGCs are active and that their activation is highly dependent on environmental conditions (e.g., nutrient availability). In terms of approach, the reconstruction of BGCs from metagenomic sequences has been carried out for numerous other ecosystems (references in the manuscript) and has revealed a significant level of novelty in each case.

However, the presentation and interpretation of the results is sometimes ambiguous and does not take full advantage of the originality of the experimental system and of the data obtained.
Thus, all expression data (from line L213 onwards) should be reported in relation to the proportions of taxa in the samples. For example, stating that L221 (but see also L247-...) “Acidobacteria expressed the majority of BGCs, followed by...” is ambiguous and can be interpreted in different ways. In fact, Acidobacteria is also the most numerically represented taxonomic group. By correcting the data for the abundance of taxa or the origin of BGCs, perhaps (??) we would see that BGCs from phylum X are statistically more active than BGCs from Acidobacteria?

However, it is the analysis of transcriptomic data that differs most from the environmental and metagenomic data obtained. At this stage, the authors pool all their samples taken at different levels of the soil core, which differ in terms of their environmental characteristics and microbial communities. In parallel, they also group MAGs, possibly from different phyla, based on the categories of genes they express. This approach is unusual, to say the least, and amounts to no longer considering microbial taxa individually, even though a great deal of bioinformatic effort had been devoted to individualizing them.
I find the results and their interpretations unconvincing. For example, it is stated on L276-277 that BGCs are particularly expressed in MAGs expressing stress responses and aerobic metabolism (Fig. 6). In this figure, it is difficult to see which “functional” categories correspond to these two “physiological states.” Should we consider that the expression of antibiotic resistance genes, motility-related genes, or stationary phase genes reflects a state of stress? This is not clear.
The results should be analyzed individually for each of the most abundant MAGs. If some are found at different levels of the soil core, comparing expression profiles at different depths could provide insights into the environmental factors that modulate BGC expression within uncultivated/uncultivable taxa.

(Remarks on code availability)

Reviewer #2

(Remarks to the Author)
The authors analyse the in-situ expression of biosynthetic gene clusters of peatland soil cores using a deeply sequenced metagenomic and metatranscriptomic dataset. They find high novelty compared to BGC databases, but also show that only a small fraction of the BGC repertoire is actively expressed. They analyse BGC expression with respect to factors such as taxonomic affiliation, BGC number in the MAG, life history strategy and expression of marker genes for certain physiological processes.

The paper is interesting, timely, and well-written. Connecting in-situ BGC expression to bacterial traits and physiological processes is an important step towards the understanding of the ecological roles of secondary metabolites. The paper provides methodological innovations that will I believe will have an impact on future publications on the topic.

I have three main points:

1. The authors state that Pseudomonadota are broadly speaking copiotrophs, while Acidobacteriota are broadly speaking oligotrophs and base a section of their discussion on this. While it is true that any randomly chosen genus of the whole phylum of Pseudomonadota is likely to be a copiotroph rather than oligotroph, I don’t think that applying this blanket statement to a set of 119 MAGs from a handful of genera is correct. There is a huge diversity of lifestyles in Pseudomonadota and especially many soil-dwelling Alphaproteobacteria are known to be oligotrophs. Therefore, I don’t think that the discussion from lines 348-375 is supported by the data. Since good-quality MAGs are available, I suggest that the authors try to estimate maximum growth rate from genomic features such as ribosomal protein codon usage bias, 16S copy number (might be difficult with short-read MAGs). Alternatively, literature inferences could be made about the families or genera present, which might prove difficult in case on uncultured genera.
2. Sampling depth is a significant variable in the study and the authors find connections between depth and e.g. BGC count per genome. It is therefore important to check the correlation of depth with other MAG statistics that could affect BGC count (completeness, N50, contig number, coverage, GC% etc) to ensure that the findings are not artifacts of e.g. lower MAG completeness.
3. The authors report that only 16.4% of BGCs were complete according to antiSMASH. Short reads tend to recover large modular BGCs such as long NRPS or PKS in fragments. Therefore, a large BGC might be recovered in multiple fragments and thus counted multiple times, which would affect e.g. the data in Fig. 2. The high coverage of the metagenomes compensates for this to a certain extent, but the concern remains. It is difficult to ascertain the magnitude of this without additional long-read sequencing. However, the authors should address this issue by e.g. checking whether there are correlations between e.g. (NRPS/PKS-) BGC number and MAG coverage or BGC number and number of contigs in a MAG; or by analysing the number of NRPS/PKS BGCs in which the main biosynthetic enzyme sits right at the edge of the contig, etc. If the authors think that it is not a concern, they should justify it in the discussion.

Other small comments, by line:

41: “Assembly-line” biosynthesis mostly refers to NRPS or T1PKS/T2PKS. Many other BGCs don’t work in an assembly-line fashion.
57-58: These sentences seem detached from the rest of the text.
112: It might be useful to include a median of completeness and contamination. Most MAGs in the study are much higher quality than the cutoff mentioned.
137-140: This sentence needs reformulating for clarity
240: I think the reference should be for Fig S7
363: It is mentioned for the first time that predicted acidobacterial metabolites are “metal chelators, siderophores, and redox-active peptides”. This should be included in the results?
477: The authors might consider also using CheckM2
503: BiG-SLiCE mode/parameters should be specified

Supplementary Figure 5: I think there is a typo here; a “>” should be a “<”

(Remarks on code availability)
I could not access the zenodo preview link – I get “permission denied” when logged in with my ORCID ID.

Version 1:

Reviewer comments:

Reviewer #1

(Remarks to the Author)
The revised version of the manuscript provides a well-argumented response to the reviewers’ comments on the first draft.

Among the main changes addressed in new sections is the description of new bioinformatic analyses of metatranscriptomic data. These analyses weight the expression levels of secondary metabolite gene clusters according to the prevalence of the bacterial phyla or MAGs with which they are associated. Expression analyses were also performed at the single MAG level, correlated with the transcription levels of different categories of functional genes and the distribution of bacterial taxa along the analyzed peatland soil core. These new analyses lead to an ecological interpretation of the data and reveal new correlations between microbial physiology and secondary metabolite synthesis, while highlighting the underestimated contribution of certain bacterial phyla to the production of these molecules. These new analyses have led to the creation of new informative figures and the removal of previously generated ones.
Overall, the study remains an original piece of work, and the revisions made reinforce this originality by highlighting the role of neglected microbial phyla and the diversity of mechanisms governing the expression of secondary metabolism genes within the bacterial kingdom.
I suggest that a few further revisions be made to the text, particularly to improve its readability.
- The authors use expressions that are sometimes difficult to understand and that should either be very clearly defined or replaced with other expressions, even if they are longer. This is the case, for example, with “in situ deployment” (L39), which appears as early as the introduction, or “BGC deployment,” which is used repeatedly. As a result, entire sentences become incomprehensible, such as the last one in the abstract: …. “in situ deployment” (?) is integrated into the “carbon-cycling activity” (?) of individual taxa rather than “a shared physiological state” (?). See also “housekeeping normalized” in several places (=”normalized with respect to housekeeping genes”?)
- In the result section, one section is entitled “In situ BGC expression” and the next one “In situ BGC expression on genome level”. Please try to clarify better the differences between these two sections as different sentences in each of them are almost identical; e.g. L278-280 +/-= L247-249, L296 = L254,......
- L237 “more than 5”, since the authors are describing MAGs with less than 10 clusters, in this case it would be advisable to give a range with exact values (e.g. 5-7).
- L250 1,120, add the percentage it represents
- Idem for L254
- Fig. 2: please make sure that the content of this figure corresponds to what is described in the text (1) I could not see a comment on panel D in the main text; (2) legend of panel B does not seem to correspond to what is shown; (3) L1016 “GCF abundance......peatland depth” is not shown in panel A
- Fig. 5: in panel C I could not see the points (in black?) corresponding to the Pseudomonadota

Reviewer #2

(Remarks to the Author)
The authors have improved their methodology. The conclusions are smaller in scope than in the first version, but they are better supported by the data. I think their approach to dealing with the fragmentation of modular BGCs is a good compromise for dealing with an issue that is inherent in short-read data.

I recommend pubication, but I have one note:

Line 156:
“This suggests that fragmentation primarily inflates absolute counts of modular BGCs and rare fragmentary families, while having less influence on the broader diversity patterns recovered at the GCF level.”
I would argue that the impact of the removal of fragmented BGCs is actually higher on GCF diversity than on the BGC counts, since GCF numbers were reduced 26%, while BGC count was only reduced 16%. The removed small NRPS/PKS fragments contributed disproportionally to the GCF richness.

Open Access This Peer Review File is licensed under a Creative Commons Attribution 4.0 International License, which permits use, sharing, adaptation, distribution and reproduction in any medium or format, as long as you give appropriate credit to the original author(s) and the source, provide a link to the Creative Commons license, and indicate if changes were made.
In cases where reviewers are anonymous, credit should be given to 'Anonymous Referee' and the source.
The images or other third party material in this Peer Review File are included in the article's Creative Commons license, unless indicated otherwise in a credit line to the material. If material is not included in the article's Creative Commons license and your intended use is not permitted by statutory regulation or exceeds the permitted use, you will need to obtain permission directly from the copyright holder.
To view a copy of this license, visit https://creativecommons.org/licenses/by/4.0/

Responses to reviewer comments:

Reviewer #1:

Major comments

This original metagenomic study has led to the identification of a significant number of new secondary metabolite synthesis gene clusters (BGCs) at different depths in peatland cores.
The strengths and originality of this study are:
- The exploration of an ecosystem that has been understudied in functional metagenomics and which is characterised by strong environmental gradients over short distances, allowing hypotheses of functional adaptation of communities to be tested.
- The reconstruction of a large number of metagenome assembled genomes (MAGs) from metagenomic data, allowing BGCs, the overwhelming majority of which are new, to be associated with bacterial phyla.
- This association reveals the significant synthesis potential of understudied and often neglected bacterial phyla, such as acidobacteria.
- The association of metagenomic data with metatranscriptomic data reveals that a minority (and which ones) of BGCs are transcriptionally active in the samples studied.

This study confirms a well-known fact acquired in the laboratory, namely that within a microbial genome, only a very limited number of BGCs are active and that their activation is highly dependent on environmental conditions (e.g., nutrient availability). In terms of approach, the reconstruction of BGCs from metagenomic sequences has been carried out for numerous other ecosystems (references in the manuscript) and has revealed a significant level of novelty in each case.

We appreciate the time that you invested in reviewing our manuscript. Please find our answers regarding your comments below.

However, the presentation and interpretation of the results is sometimes ambiguous and does not take full advantage of the originality of the experimental system and of the data obtained.
Thus, all expression data (from line L213 onwards) should be reported in relation to the proportions of taxa in the samples. For example, stating that L221 (but see also L247-...) "Acidobacteria expressed the majority of BGCs, followed by..." is ambiguous and can be interpreted in different ways. In fact, Acidobacteria is also the most numerically represented taxonomic group. By correcting the data for the abundance of taxa or the origin of BGCs, perhaps (??) we would see that BGCs from phylum X are statistically more active than BGCs from Acidobacteria?

We thank the reviewer for this important point. We agree that our original wording could conflate total community contribution with phylum-specific biosynthetic activity, because highly abundant taxa may contribute strongly to the BGC transcript pool simply by being abundant. In the revised manuscript, we therefore separate raw contribution from abundance-normalized and inventory-normalized measures of BGC expression.

First, at the contig level, we now report phylum-level contributions as the share of total BGC transcript abundance and the number of expressed BGCs contributed by each phylum, normalized by the metagenome abundance for each of these phyla (**Lines 261 - 275**) and **Fig 4c,d**.

Second, in the MAG-level BGC expression analyses, we now account for differences in taxon abundance using normalized phylum-level BGC expression relative to metagenome-level phylum relative abundance at the corresponding depth. This allowed us to identify phyla whose contribution to BGC transcription exceeded their proportional representation in the community (**Lines 305 - 321**). We report mean BGC expression intensity, i) as the mean TPM per active BGC (**Supplementary Fig. S9a**), ii) as phylum-level metagenome abundance normalized BGC expression (**Supplementary Fig. S9b**), and iii), at the genome level, we normalized expression by biosynthetic inventory by quantifying the fraction of encoded BGCs expressed per MAG and summarizing these deployment rates across phyla (**Fig 5d**).

These complementary analyses distinguish whether a phylum contributes strongly to BGC expression because it is abundant in the community, because it encodes many BGCs, or because it deploys a larger fraction of its encoded biosynthetic repertoire. We have revised the results section (**Lines 261 - 275**, **Lines 305 - 321**) accordingly and added the corresponding normalized analyses to **Fig. 4c**,**d**, **Fig. 5d** and **Supplementary Fig. S9**.

However, it is the analysis of transcriptomic data that differs most from the environmental and metagenomic data obtained. At this stage, the authors pool all their samples taken at different levels of the soil core, which differ in terms of their environmental characteristics and microbial communities. In parallel, they also group MAGs, possibly from different phyla, based on the categories of genes they express. This approach is unusual, to say the least, and amounts to no longer considering microbial taxa individually, even though a great deal of bioinformatic effort had been devoted to individualizing them.

This point was addressed in detail, along with the comment below.

I find the results and their interpretations unconvincing. For example, it is stated on L276-277 that BGCs are particularly expressed in MAGs expressing stress responses and aerobic metabolism (Fig. 6). In this figure, it is difficult to see which "functional" categories correspond to these two "physiological states." Should we consider that the expression of antibiotic resistance genes, motility-related genes, or stationary phase genes reflects a state of stress? This is not clear.

The results should be analyzed individually for each of the most abundant MAGs. If some are found at different levels of the soil core, comparing expression profiles at different depths could provide insights into the environmental factors that modulate BGC expression within uncultivated/uncultivable taxa.

We thank the reviewer for raising this important criticism. We agree that our original analysis pooled transcriptomic data and functional categories too broadly, thereby weakening the link between the MAG-resolved dataset and the interpretation. In particular, we agree that AMR, motility, chemotaxis, stationary-phase markers, and other functional categories should not be interpreted together as a single "stress response" or as evidence for one shared physiological state.

We have therefore substantially revised this section and reanalyzed the transcriptomic data at the individual MAG × depth level, as suggested. In the revised **Fig. 6**, 15 focal, BGC-encoding MAGs are shown individually across three depth intervals, with depth information retained throughout. These MAGs were selected to capture the main contributors to BGC core-gene expression and MAGs showing detectable BGC deployment across depths. The revised figure now reports the percentage and number of expressed BGCs per product class for each MAG and depth, together with selected metabolic features related to aerobic/microaerobic respiration, redox-associated and anaerobic energy metabolism, fermentation and carbon processing.

Additional KEGG-derived trait modules, including resource acquisition, stress resistance, and interaction/defence markers, are now shown separately in the newly added **Supplementary Fig. 12**. This allows expression profiles to be compared directly within individual MAGs across depths, rather than grouping MAGs from different phyla into broad physiological categories.

This reanalysis led us to revise the interpretation. We no longer conclude that BGC expression is generally associated with a common "stress response" or "aerobic metabolism" state. Instead, the revised MAG-resolved analysis shows that BGC-expressing MAGs occur across a mosaic of taxon- and depth-specific metabolic contexts. Some MAGs expressed terminal oxidases, others showed anaerobic-energy-associated functions, and others showed stronger carbon-processing features, but no single physiological state was shared across all BGC-active MAGs. Across this heterogeneity, the most consistent signal was a positive association between BGC expression and carbohydrate-active enzyme expression, particularly glycoside hydrolase expression, which was supported by mixed-effects modeling and co-deployment analyses. We also have revised the text in results (**Lines 351 - 406**) and discussion (**L466 - 471**, **L507 – L518**, **L519 – L524**) to present this more conservative MAG interpretation.

Reviewer #2:

The authors analyse the in-situ expression of biosynthetic gene clusters of peatland soil cores using a deeply sequenced metagenomic and metatranscriptomic dataset. They find high novelty compared to BGC databases, but also show that only a small fraction of the BGC repertoire is actively expressed. They analyse BGC expression with respect to factors such as taxonomic affiliation, BGC number in the MAG, life history strategy and expression of marker genes for certain physiological processes.

The paper is interesting, timely, and well-written. Connecting in-situ BGC expression to bacterial traits and physiological processes is an important step towards the understanding of the ecological roles of secondary metabolites. The paper provides methodological innovations that will I believe will have an impact on future publications on the topic.

Thank you for your kind words. One of our main motivations was to go beyond the mere cataloging of BGCs. We believe that, while our approach has inherent shortcomings and limitations, it can be helpful to other groups studying BGCs in the environment. Consequently, we decided to share all the used code, including comprehensive descriptions of what was done.

Major comments

1. The authors state that Pseudomonadota are broadly speaking copiotrophs, while Acidobacteriota are broadly speaking oligotrophs and base a section of their discussion on this. While it is true that any randomly chosen genus of the whole phylum of Pseudomonadota is likely to be a copiotroph rather than oligotroph, I don't think that applying this blanket statement to a set of 119 MAGs from a handful of genera is correct. There is a huge diversity of lifestyles in Pseudomonadota and especially many soil-dwelling Alphaproteobacteria are known to be oligotrophs. Therefore, I don't think that the discussion from lines 348-375 is supported by the data. Since good-quality MAGs are available, I suggest that the authors try to estimate maximum growth rate from genomic features such as ribosomal protein codon usage bias, 16S copy number (might be difficult with short-read MAGs). Alternatively, literature inferences could be made about the families or genera present, which might prove difficult in case on uncultured genera.

We thank the reviewer for this important point. We agree that our original discussion relied too heavily on broad phylum-level trophic labels, particularly for Pseudomonadota, which encompass a wide range of lifestyles and include many soil-dwelling Alphaproteobacteria that do not fit a simple copiotroph classification. We have therefore substantially revised this section and no longer treat Acidobacteriota and Pseudomonadota as uniformly oligotrophic or copiotrophic groups.

In response to the reviewer's suggestion, we estimated growth-related traits for the recovered MAGs using codon-usage-bias-based predicted doubling times (**L333 - 346**, **Supplementary Fig. 11**). Predicted doubling times differed significantly between Acidobacteriota and Pseudomonadota, with Pseudomonadota tending toward lower values overall. This pattern was also apparent among BGC-rich lineages, where many Pseudomonadota families with ≥5 BGCs had lower mean predicted doubling times than comparably BGC-rich Acidobacteriota families. We now present these results as evidence for differences in predicted growth potential among the recovered MAGs, rather than as categorical phylum-level lifestyle assignments (**L 337**).

We also compared carbon-processing traits because these were more directly linked to BGC expression in our dataset. Acidobacteriota encoded more CAZymes per genome and showed higher expression of carbohydrate-active enzymes, particularly glycoside hydrolases (**Supplementary Fig 11c, d**, **L334 - 349**). Across MAGs, the fraction of expressed BGCs was strongly positively associated with glycoside hydrolase expression and total CAZyme expression, and this relationship remained significant in a binomial mixed-effects model accounting for repeated genomes and samples. We therefore revised the Discussion **(Line 457 – 458, Line 472 – 478, Line 486 - 492**) to frame the Acidobacteriota–Pseudomonadota contrast as a difference in measured traits, mainly predicted growth potential, CAZyme repertoire, and carbohydrate-active expression, rather than as a strict copiotroph-oligotroph dichotomy.

2. Sampling depth is a significant variable in the study and the authors find connections between depth and e.g. BGC count per genome. It is therefore important to check the correlation of depth with other MAG statistics that could affect BGC count (completeness, N50, contig number, coverage, GC% etc) to ensure that the findings are not artifacts of e.g. lower MAG completeness.

Thank you for raising another very important point. To test whether the observed depth-related patterns in BGC content could be explained by MAG quality or assembly statistics, we compared completeness, contamination, contig N50, total contig number, genome size and GC content across peat depths **(Methods, L682 – 688)**. Contamination, contig N50 and total contig number did not differ significantly among depths (**Supplementary Fig. 2**). Completeness was slightly higher in deeper layers, whereas genome size was slightly smaller with deeper peat, and has been added to the results in **Lines 179 - L189** and in **Supplementary Fig. 2**. The decline in BGC content in deeper peat is unlikely to be caused by poorer MAG quality and if anything, completeness trends in the opposite direction.

To further account for variation in genome size, we normalized BGC content by genome length and now report BGC density as BGCs per Mbp in Fig. 2c,d. This genome-size-normalized measure also decreased with depth, supporting the conclusion that the depth-related decline in biosynthetic capacity is not an artifact of MAG completeness, fragmentation, or genome-size differences. GC content also varied with depth, which we interpret as reflecting the depth-related shift in taxonomic composition. We have added these analyses to **Supplementary Fig. 2** and revised the results section accordingly **(Line 187)**.

3. The authors report that only 16.4% of BGCs were complete according to antiSMASH. Short reads tend to recover large modular BGCs such as long NRPS or PKS in fragments. Therefore, a large BGC might be recovered in multiple fragments and thus counted multiple times, which would affect e.g. the data in Fig. 2. The high coverage of the metagenomes compensates for this to a certain extent, but the concern remains. It is difficult to ascertain the magnitude of this without additional long-read sequencing. However, the authors should address this issue by e.g. checking whether there are correlations between e.g. (NRPS/PKS-) BGC number and MAG coverage or BGC number and number of contigs in a MAG; or by analysing the number of NRPS/PKS BGCs in which the main biosynthetic enzyme sits right at the edge of the contig, etc. If the authors think that it is not a concern, they should justify it in the discussion.

You are absolutely right. Short-read assemblies can fragment large modular BGCs, particularly NRPS and PKS clusters, and this may inflate raw BGC counts if fragments of the same locus are recovered as separate antiSMASH regions. We have therefore added an explicit fragmentation-sensitivity analysis focused on modular BGC classes most vulnerable to this issue.

Following the reviewer’s suggestion, we assessed the position of biosynthetic core genes relative to contig ends. Rather than discarding all incomplete BGCs, we applied an anchored-core framework in which incomplete NRPS, PKS and NRPS-PKS hybrid BGCs were classified as weak fragments when they occurred at a contig edge and lacked any biosynthetic core gene located more than 5 kb from a contig end. This criterion was designed to identify likely edge fragments while retaining incomplete BGCs that still contained a biosynthetic core gene positioned within the contig, since incompleteness alone does not necessarily indicate that a region is a trivial assembly fragment. These details have been added to the methods in **Lines 616 - 626**.

Using this approach, we identified 1,544 of 9,694 BGCs (15.9%) as weak fragments (**Line 144**) potentially contributing to count inflation. As expected, this effect was concentrated in modular classes, affecting 41.5% of NRPS BGCs, 39.2% of PKS BGCs and 20.5% of NRPS-PKS hybrid BGCs. However, many contig-edge modular BGCs retained anchored biosynthetic core genes and were therefore retained, including 1,217 NRPS, 953 PKS and 260 NRPS-PKS hybrid BGCs. Thus, fragmentation affects absolute counts of modular BGCs, but does not indicate a dataset-wide overestimation of biosynthetic diversity.

To evaluate whether this altered family-level conclusions, we combined the anchored-core filtering with our BiG-SCAPE GCF assignments. Excluding weak fragments reduced both raw BGC counts and GCF-level richness. We now describe this limitation explicitly in the revised manuscript, report the anchored-core sensitivity analysis in **Lines 141-161**, and clarify that filtered GCF counts should be interpreted as conservative lower-bound sensitivity estimates rather than

corrected “true” richness values. The data in **Fig. 2ab** has also been updated to reflect the GCFs created from fragmentation-filtered BGCs.

Minor comments:

41: “Assembly-line” biosynthesis mostly refers to NRPS or T1PKS/T2PKS. Many other BGCs don’t work in an assembly-line fashion.

We thank the reviewer for catching this error. We have rephrased the sentence and removed the mention of “assembly-line” in **Line 43**.

57-58: These sentences seem detached from the rest of the text.

The sentences in question have been altered to more coherent concluding sentences to this paragraph, which flows with the topic of the rest of the paragraph and the next paragraph. The revised text appears in **Lines 58 - 62** of the revision.

112: It might be useful to include a median of completeness and contamination. Most MAGs in the study are much higher quality than the cutoff mentioned.

We thank the reviewer for this suggestion, because the median MAG stats (median completeness of 94.4 % and contamination of 2.2%), added in **Lines 118 - 119** supersede medium MIMAG-quality standards. We have also added a figure with MAG stats and cited it along with adding median completeness and contamination to the results section (**Supplementary Fig. 2**).

137-140: This sentence needs reformulating for clarity

We have clarified the sentence in question, partly by reducing the amount of detail in **Lines 155 - 161**.

240: I think the reference should be for Fig S7

The text has been corrected accordingly, and the reference is now to the correct figure.

363: It is mentioned for the first time that predicted acidobacterial metabolites are “metal chelators, siderophores, and redox-active peptides”. This should be included in the results?

Correct. This statement was removed during revision of the discussion in response to the reviewer comment regarding physiological states, stress, and BGC expression above.

477: The authors might consider also using CheckM2

We thank the reviewer for this useful suggestion. In preparation for other MAG-quality related comments above we rerun our MAG dataset using CheckM2. The manuscript has been updated accordingly.

503: BiG-SLiCE mode/parameters should be specified

We have clarified that we used the ‘query’ function against the BGC-Atlas database, and performed our own clustering, followed by ‘query’ against MiBiG v4 with input parameters provided where necessary. These details have been added to the methods section in **Lines 637 - 640**.

Supplementary Figure 5: I think there is a typo here; a “>” should be a “<”

Good catch. We have fixed the error. The figure in question is now **Supplementary Fig. 6**.

I could not access the zenodo preview link – I get "permission denied" when logged in with my ORCID ID.

We apologize for this error, likely due to the long reviewer link provided by Zenodo. We have created a shortened link here: https://tinyurl.com/PeatlandBGCs and we have confirmed that the complete link works:

https://zenodo.org/records/17812908?preview=1&token=eyJhbGciOiJIUzUxMiJ9.eyJpZCI6ImRkYjBlYmMxLWVhN2QtNDYyMS1hYzhkLTA0N2IwMjI2MGMxOSIsImRhdGEiOnt9LCJyYW5kb20iOiJiNTVkOTFlNTQ2ZTMwOGQwMzI4NjA4Y2NhYzU2MjU4YiJ9.avEKbnn-POY_ZrM3w7VJy2WIGLbeZlyOXiuckjFNdfiV3q3P6Fe6VCMvoEb-j9vQka79zU_HD4uta_w0xbOqOg

Reviewer 1

The revised version of the manuscript provides a well-argumented response to the reviewers' comments on the first draft. Among the main changes addressed in new sections is the description of new bioinformatic analyses of metatranscriptomic data. These analyses weight the expression levels of secondary metabolite gene clusters according to the prevalence of the bacterial phyla or MAGs with which they are associated. Expression analyses were also performed at the single MAG level, correlated with the transcription levels of different categories of functional genes and the distribution of bacterial taxa along the analyzed peatland soil core. These new analyses lead to an ecological interpretation of the data and reveal new correlations between microbial physiology and secondary metabolite synthesis, while highlighting the underestimated contribution of certain bacterial phyla to the production of these molecules. These new analyses have led to the creation of new informative figures and the removal of previously generated ones.

Overall, the study remains an original piece of work, and the revisions made reinforce this originality by highlighting the role of neglected microbial phyla and the diversity of mechanisms governing the expression of secondary metabolism genes within the bacterial kingdom.
I suggest that a few further revisions be made to the text, particularly to improve its readability.

We thank the reviewer for their careful reading of the revised manuscript and for their positive assessment of the new analyses. We are grateful for the further comments on readability, which we address below.

- The authors use expressions that are sometimes difficult to understand and that should either be very clearly defined or replaced with other expressions, even if they are longer. This is the case, for example, with "in situ deployment" (L39), which appears as early as the introduction, or "BGC deployment," which is used repeatedly. As a result, entire sentences become incomprehensible, such as the last one in the abstract: …. "in situ deployment" (?) is integrated into the "carbon-cycling activity" (?) of individual taxa rather than "a shared physiological state" (?). See also "housekeeping normalized" in several places (="normalized with respect to housekeeping genes"?)

We agree that this terminology was unnecessarily unclear and thank the reviewer for identifying it. In most instances 'deployment' simply referred to expression in the environment, and we have replaced it with 'BGC expression' throughout. (**L39, L330, L364, L406, L407, L410, L411, L429, L749 and L751**).

In the paragraph starting at **L405**, deployment carried a different meaning: the fraction of genome-encoded BGCs that are expressed. Here it has been corrected to:

"We further tested whether this association was also present at the level of BGC deployment evident in the fraction of genome-encoded BGCs expressed (as opposed to intensity of transcription / TPM above). The proportion of genome-encoded BGC expressed varied strongly among MAGs…".

The reviewer's interpretation of "housekeeping-normalized" is correct, and we agree that this was also unclear. We have replaced it with 'normalised to the mean TPM of single-copy housekeeping genes' in **L374**, **L391**, and the legend of Fig 6C (**L1133**).

- In the result section, one section is entitled "In situ BGC expression" and the next one "In situ BGC expression on genome level". Please try to clarify better the differences between these two sections as different sentences in each of them are almost identical; e.g. L278-280 +/-= L247-249, L296 = L254,......

We thank the reviewer for noting this redundancy. The two sections address expression at the contig/assembly level and at the genome level, respectively, and the headings did not convey this. We have renamed the first section "Community-wide BGC transcription" (**L254**) and the second "In situ expression of BGC repertoires in individual genomes" (**L287**). We have also removed the duplicated sentences at L278 -280 and L296 in the previous version of the manuscript, retaining each statement only where it is relevant and provided more detail to make these sections easily distinguishable.

- L237 "more than 5", since the authors are describing MAGs with less than 10 clusters, in this case it would be advisable to give a range with exact values (e.g. 5-7).

We agree that this phrasing was imprecise. We have rewritten this paragraph to make it clearer, provide more relevant details and specify the ranges of BGCs as suggested by the reviewer (**L243-L246**).

- L250 1,120, add the percentage it represents

We have added the percentage represented by the number of expressed BGCs as suggested by the reviewer (13.7%) (**L259**).

- Idem for L254

We added the respective percentages here as well (**L264-L265**).

- Fig. 2: please make sure that the content of this figure corresponds to what is described in the text (1) I could not see a comment on panel D in the main text; (2) legend of panel B does not seem to correspond to what is shown; (3) L1016 "GCF abundance......peatland depth" is not shown in panel A

We thank the reviewer for catching this error. We have added the missing text referring to Fig 2D. We have also fixed Fig 2, and updated the legend to correspond to the correct panel.

- Fig. 5: in panel C I could not see the points (in black?) corresponding to the Pseudomonadota

We thank the reviewer for catching this detail, which refers to Fig 6C. We have corrected the figure and the legend which reflects the correct data.

Reviewer #2 (Remarks to the Author):

The authors have improved their methodology. The conclusions are smaller in scope than in the first version, but they are better supported by the data. I think their approach to dealing with the fragmentation of modular BGCs is a good compromise for dealing with an issue that is inherent in short-read data.

I recommend publication, but I have one note:

Line 156:
"This suggests that fragmentation primarily inflates absolute counts of modular BGCs and rare fragmentary families, while having less influence on the broader diversity patterns recovered at the GCF level."
I would argue that the impact of the removal of fragmented BGCs is actually higher on GCF diversity than on the BGC counts, since GCF numbers were reduced 26%, while BGC count was only reduced 16%. The removed small NRPS/PKS fragments contributed disproportionally to the GCF richness.

We agree. The removal of fragmented BGCs had a greater effect on GCF richness than on total BGC counts. We have revised the text to clarify that fragmented, particularly NRPS and PKS NGCs disproportionately contributed to rare GCFs (**L155-L158**).
